# Type VI Secretion System Transports Zn^2+^ to Combat Multiple Stresses and Host Immunity

**DOI:** 10.1371/journal.ppat.1005020

**Published:** 2015-07-02

**Authors:** Tietao Wang, Meiru Si, Yunhong Song, Wenhan Zhu, Fen Gao, Yao Wang, Lei Zhang, Weipeng Zhang, Gehong Wei, Zhao-Qing Luo, Xihui Shen

**Affiliations:** 1 State Key Laboratory of Crop Stress Biology for Arid Areas and College of Life Sciences, Northwest A&F University, Yangling, Shaanxi, China; 2 Department of Biological Sciences, Purdue University, West Lafayette, Indiana, United States of America; Vanderbilt University, UNITED STATES

## Abstract

Type VI secretion systems (T6SSs) are widespread multi-component machineries that translocate effectors into either eukaryotic or prokaryotic cells, for virulence or for interbacterial competition. Herein, we report that the T6SS-4 from *Yersinia pseudotuberculosis* displays an unexpected function in the transportation of Zn^2+^ to combat diverse stresses and host immunity. Environmental insults such as oxidative stress induce the expression of T6SS-4 via OxyR, the transcriptional factor that also regulates many oxidative response genes. Zinc transportation is achieved by T6SS-4-mediated translocation of a novel Zn^2+^-binding protein substrate YezP (YPK_3549), which has the capacity to rescue the sensitivity to oxidative stress exhibited by T6SS-4 mutants when added to extracellular milieu. Disruption of the classic zinc transporter ZnuABC together with T6SS-4 or *yezP* results in mutants that almost completely lost virulence against mice, further highlighting the importance of T6SS-4 in resistance to host immunity. These results assigned an unconventional role to T6SSs, which will lay the foundation for studying novel mechanisms of metal ion uptake by bacteria and the role of this process in their resistance to host immunity and survival in harmful environments.

## Author Summary

One unique feature of type VI secretion system is the presence of multiple distinct systems in certain bacterial species. It is well established that some of these systems function to compete for their living niches among diverse bacterial species, whilst the activity of many such transporters remains unknown. Because metal ions are essential components to virtually all forms of life including bacteria, eukaryotic hosts have evolved complicated strategies to sequester metal ions, which constitute a major branch of their nutritional immunity. Therefore the ability to acquire metal ions is critical for bacterial virulence. This study reveals that the T6SS-4 of *Yersinia pseudotuberculosis* (*Yptb*) functions to import Zn^2+^ from the environment to mitigate the detrimental effects such as hydroxyl radicals induced by diverse stresses. Expression of the transporter is activated by multiple regulatory proteins, including OxyR and OmpR that sense diverse environmental cues. Zinc ion acquisition is achieved by translocating a Zn^2+^-binding substrate YezP, which is co-regulated with T6SS-4 by OxyR. Our results reveal a novel role for type VI secretion system, which is important in the study of the mechanism of metal ion acquisition by bacteria and the role of this process in bacterial pathogenesis and survival in detrimental environments.

## Introduction

Specialized protein secretion systems are essential for many bacteria to survive in interactions with their hosts or within specific environmental niches. Among these, type VI secretion system (T6SS) is a complex macromolecular apparatus found in more than 25% of sequenced Gram-negative bacteria genomes, ranging from pathogens to environmental species [1]. Structurally related to the contractile phage tail sheath, the T6SS is composed of 13 conserved proteins and a variable array of accessory elements [1,2]. The extracellular components of the T6SS, Hcp (hemolysin-coregulated protein) and VgrG (valine glycine repeat) form a needle-like injection device closely resembling the bacteriophage tail, in which VgrG forms a cell-puncturing tip, and Hcp forms a tail-tube structure through which effector proteins are believed to travel [1,2]. ClpV and IcmF, two conserved components with ATPase activity that powers the T6SS, are crucial for the secretion of Hcp, VgrG and its cognate protein substrates [1,2].

A striking feature of T6SS is that many genomes harbor multiple gene clusters coding for evolutionarily distinct T6SSs, which presumably play different roles in the lifecycle of the bacteria [1,3]. Several T6SSs associated with pathogens are necessary for full virulence towards eukaryotic host cells [4,5]. In *Vibrio cholera*, T6SS is required for virulence in animal infection or for resistance to predation by amoebae hosts such as *Dictyostelium discoideum* [4,5]. In *Burkholderia mallei*, the cluster 1 T6SS expressed by this organism is essential for bacterial survival in a hamster model of glanders and the *tssE* mutants exhibit growth and actin polymerization defects in RAW 264.7 murine macrophages [6]. On the contrary, some T6SSs appear to be antivirulence factors because mutants lacking such systems are more pathogenic [7,8]. Deletion of the T6SS in *Helicobacter hepaticus* led to mutants that adhere and enter epithelial cell at high efficiencies than wild-type bacteria [5]. In these scenarios, effectors, the T6SS apparatus or its components may stimulate the host immune response to suppress the virulence of wild-type bacteria.

The best-characterized function of T6SSs is to compete in bacterial communities by delivering bacteriolytic toxins to target cells [2,9]. For example, a T6SS in *Pseudomonas aeruginosa* delivers at least two families of effectors into target bacterial cells, which function as peptidoglycan hydrolases and phospholipase, respectively [9,10]. These effectors mediate antagonistic bacterial interactions in either inter- or intraspecies context to gain a survival advantage in specific niches. Similarly, *Agrobacterium tumefaciens* uses T6SS to translocate antibacterial DNases to attack neighboring bacterial cells in plant hosts [11]. Interestingly, in each case, the toxicity of the effectors toward the bacterial cell itself is inhibited by specific immunity proteins, which directly interact with the effectors [9,11]. Roles of T6SSs in biological processes beyond infection and inter-species competition have also been suggested [12–14], but little is known about the underlying mechanisms.

Whereas the genomes of many bacteria harbor one to two T6SS gene clusters [1], the closely related *Yersinia pseudotuberculosis* (*Yptb*) and *Yersinia pestis* contain four and five such clusters, respectively [1]. These systems likely confer distinct functions for specific niches in the lifecycle of the bacterium, thus representing excellent models for the study of the potentially versatile function of T6SSs. Here we found that the T6SS-4 of *Yptb* functions to acquire zinc ions (Zn^2+^) into bacterial cells from the environment, which mitigates the hydroxyl radicals induced by oxidative stresses. Our results reveal that diverse environmental insults activate the expression of T6SS-4 via OxyR, the primary regulatory protein for bacterial oxidative stress and that zinc acquisition is achieved by T6SS-4-mediated translocation of a zinc-binding protein into the extracellular milieu. While it is well established that when appropriately deployed, some T6SSs confer the bacterium surviving advantages in niches with multiple bacterial species by delivering bacteriolytic toxins to competing cells, our results uncover a novel function of T6SS in the acquisition of essential nutrients, which enhances bacterial survival under harsh environments and/or during its interactions with hosts.

## Results

### Expression of T6SS-4 in *Yptb* is activated by OxyR

**Fig 1:**
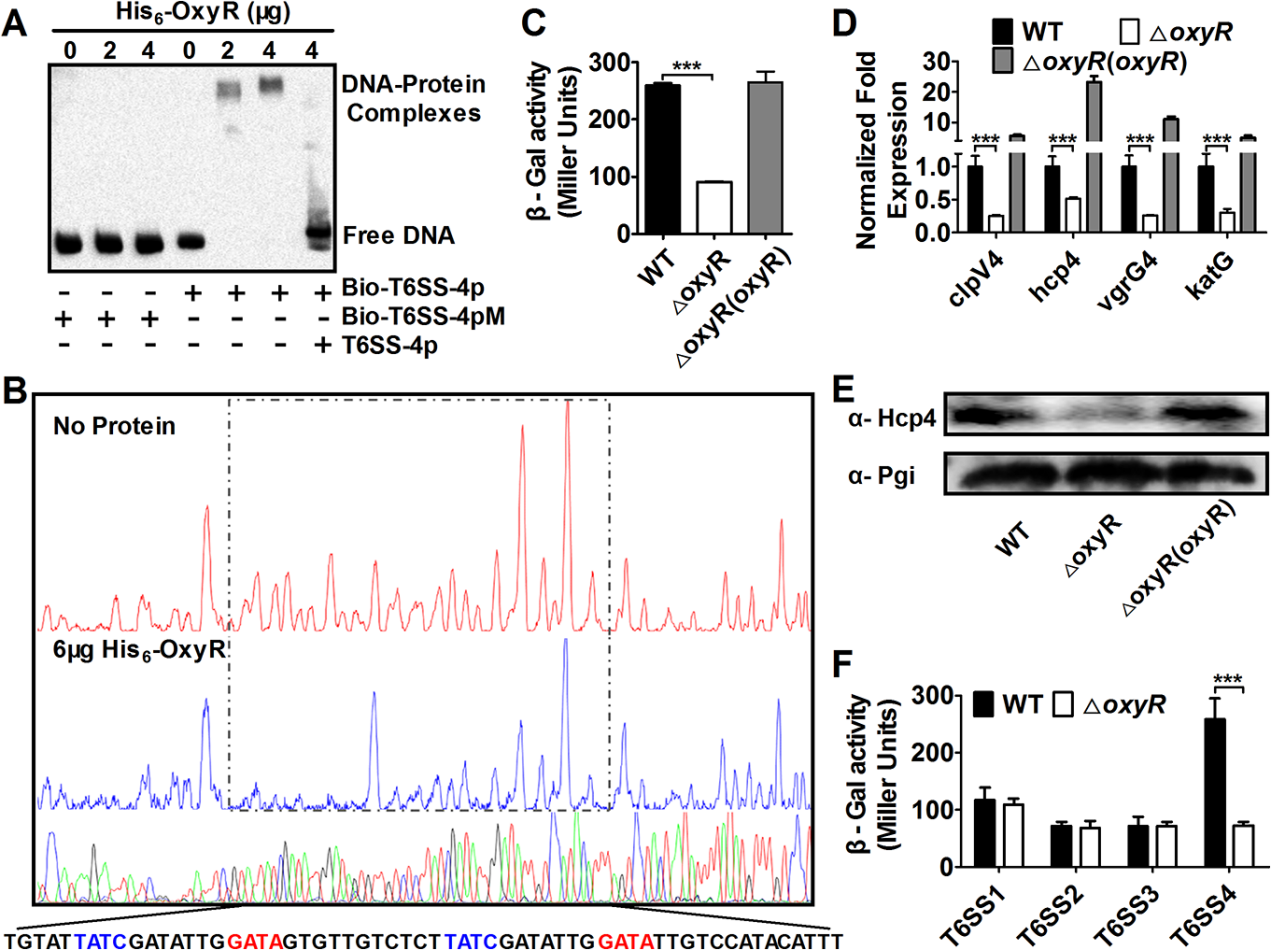
OxyR directly activates T6SS-4 expression. **A.** OxyR binds the T6SS-4 promoter. Biotin-labeled probe or its mutant was incubated with OxyR. The protein-DNA complexes were detected by streptavidin-conjugated HRP and chemiluminescent substrate. Unlabeled promoter was added to determine the binding specificity of OxyR. Bio-T6SS-4p: biotin-labeled T6SS-4 promoter. Bio-T6SS-4pM: biotin-labeled T6SS-4 promoter mutant. **B.** Identification of the OxyR protected region in T6SS-4 promoter region. Complexes formed between FAM dye-labeled probes and His_6_-OxyR were subjected to DNase I digestion. DNA was sequenced and the 4 nucleotides marked in different colors were merged. The electropherograms were aligned using GeneScan-LIZ500. **C-D.** OxyR activates the expression of T6SS-4. **β**-galactosidase activity (**C**) or relative expression measured by quantitative RT-PCR in indicated bacterial strains was determined. Relative levels of transcripts were presented as the mean values ± SD calculated from three sets of independent experiments (**D**). **E.** The protein level of Hcp4 in relevant *Yptb* strains. Lysates from bacteria were resolved by SDS-PAGE, and Hcp4 was detected by immunoblotting. The metabolic protein phosphoglucose isomerase (Pgi) was probed as a loading control. **F**. OxyR does not activate T6SS1-3. **β**-galactosidase activity from chromosomal *lacZ* fusions in relevant *Yptb* was measured. Data shown were the average of three independent experiments; error bars indicate SD from three independent experiments. ***, *p*<0.001.

To determine the function of the T6SS-4 in *Yptb*, we analyzed its promoter region and identified a DNA element highly similar to the recognition site for OxyR, the primary regulatory protein for bacterial oxidative stress (S1 Fig). We then examined the interaction between OxyR and this putative operator by electrophoresis mobility shift assay (EMSA). Incubation of a probe containing the T6SS-4 promoter with His_6_-OxyR led to the formation of DNA-protein complexes (Fig 1A). The interactions between His_6_-OxyR and the T6SS-4 promoter are specific because excessive unlabeled probe abolished the formation of the protein-DNA complex; similarly, mutations in the predicted OxyR binding site disrupted the formation of such complexes (Fig 1A). DNase I footprinting analysis revealed that the putative OxyR binding site was protected from digestion in DNA-OxyR complexes, further indicating the recognition of this DNA element by OxyR (Fig 1B). Thus, OxyR specifically recognizes an operator within the T6SS-4 promoter, most likely to influence its activity.

Next we determined the effects of OxyR on the expression of the T6SS-4 by measuring the transcription of chromosomal *P*_T6SS-4_::*lacZ* fusions. Deletion of *oxyR* significantly reduced the activity of the promoter, which can be fully restored by a complementation plasmid expressing the regulatory protein (Fig 1C). Consistent with the operon-like organization of the T6SS-4 structural genes, qRT-PCR analyses revealed that the expression of other T6SS-4 components such as *clpV4* (*ypk*_3559), *hcp4* (*ypk*_3563) and *vgrG4* (*ypk*_3558), also required OxyR, in a manner highly similar to *katG* (*ypk*_3388), one of the established target genes of this regulatory protein (Fig 1D). Further analysis at protein level indicated that similar regulation was observed for protein production of *hcp4*, in which deletion of OxyR diminished its cellular level (Fig 1E). In contrast, the expression of T6SS1-3 in *Yptb* was not detectably affected by the deletion of *oxyR*, pointing to the specificity of the regulation (Fig 1F). Thus, OxyR specifically activates the expression of T6SS-4, suggesting that its function is relevant to the environmental cues sensed by this regulatory protein.

### T6SS-4 is required for bacterial resistance to oxidative stress

**Fig 2:**
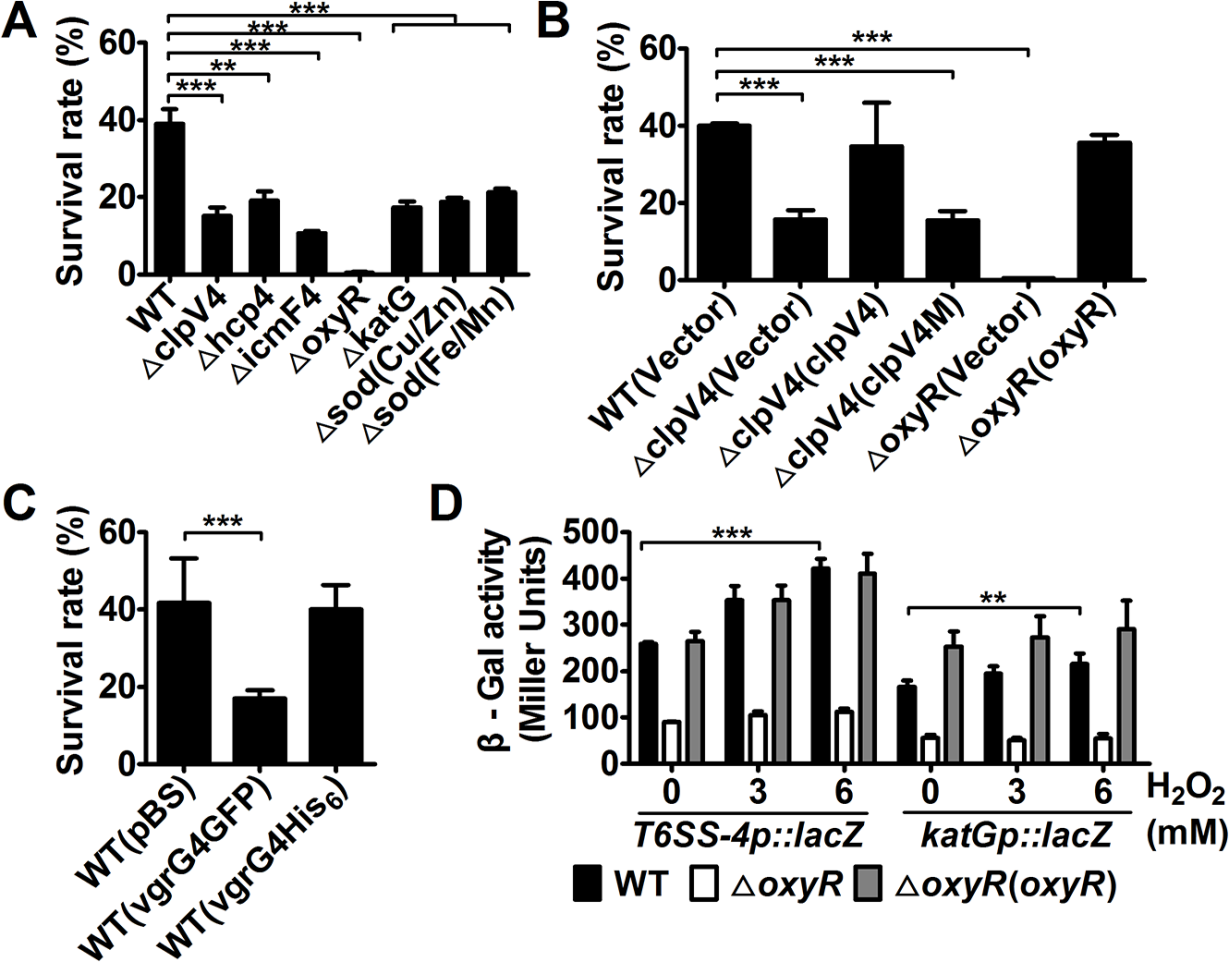
T6SS-4 is essential for *Yptb* survival under oxidative stress. **A-C**. Indicated bacterial strains grown to mid-exponential phase were exposed to H_2_O_2_ for 1 hour and the viability of the cells was determined. Note that the *clpV4M* mutant defective in ATPase activity failed to complement the *clpV4* mutant. **D**. Oxidative stress induced the expression of T6SS-4. Cells of relevant *Yptb* strains harboring *P*_T6SS-4_::*lacZ* or *P*_*katG*_::*lacZ* were treated with indicated amounts of H_2_O_2_ and the expression of the reporter was measured. Data shown were the average of three independent experiments; error bars indicate SD from three independent experiments. ***, *p*<0.001; **, *p*<0.01.

OxyR is a global oxidative stress regulator that controls the expression of genes such as *katG*, *gorA*, *grxA*, *ahpCF* and *oxyS*, all important in protection against oxidative stress [15]. The activation of T6SS-4 by OxyR prompted us to examine whether this transporter plays a role in protection against oxidative stress. We thus determined the viability of *Yptb* T6SS-4 mutants after H_2_O_2_ challenge. As a control, the *ΔkatG* mutant was expectedly sensitive to H_2_O_2_, so were the mutants defective in the Cu/Zn or Fe/Mn superoxide dismutase (SOD) (Fig 2A). The *ΔoxyR* mutant and mutants lacking essential T6SS-4 structural genes are significantly more sensitive to H_2_O_2_ than wild-type bacteria (Fig 2A). For example, about 39.0% wild-type bacteria survived after exposing to H_2_O_2_ for 1 hr, but the survival rates for the *ΔicmF4* mutant were only 10.7% (Fig 2A). Further, the sensitivity of the *ΔclpV4* mutant to oxidative stress can be fully alleviated by expressing wild-type gene but not the E^304^A/E^677^A mutant deficient in hydrolyzing ATP (Fig 2B), supporting a role of T6SS-4 in combating oxidative stress. Importantly, the conductance of the T6SS-4 channel is required for such resistance as the VgrG4-GFP fusion known to block T6SS secretion [13] rendered the bacteria sensitive to H_2_O_2_; such inhibition did not occur when VgrG4 was fused to 6 tandem histidine residues (VgrG4-His_6_), a fusion known not to block the function of the transporter [13] (Fig 2C). In agreement with these observations, similar to *katG*, one of the classical target genes of OxyR, the expression of T6SS-4 was induced by oxidative stress (Fig 2D).

### Hydroxyl radicals were accumulated in T6SS-4 mutants under stress conditions

**Fig 3:**
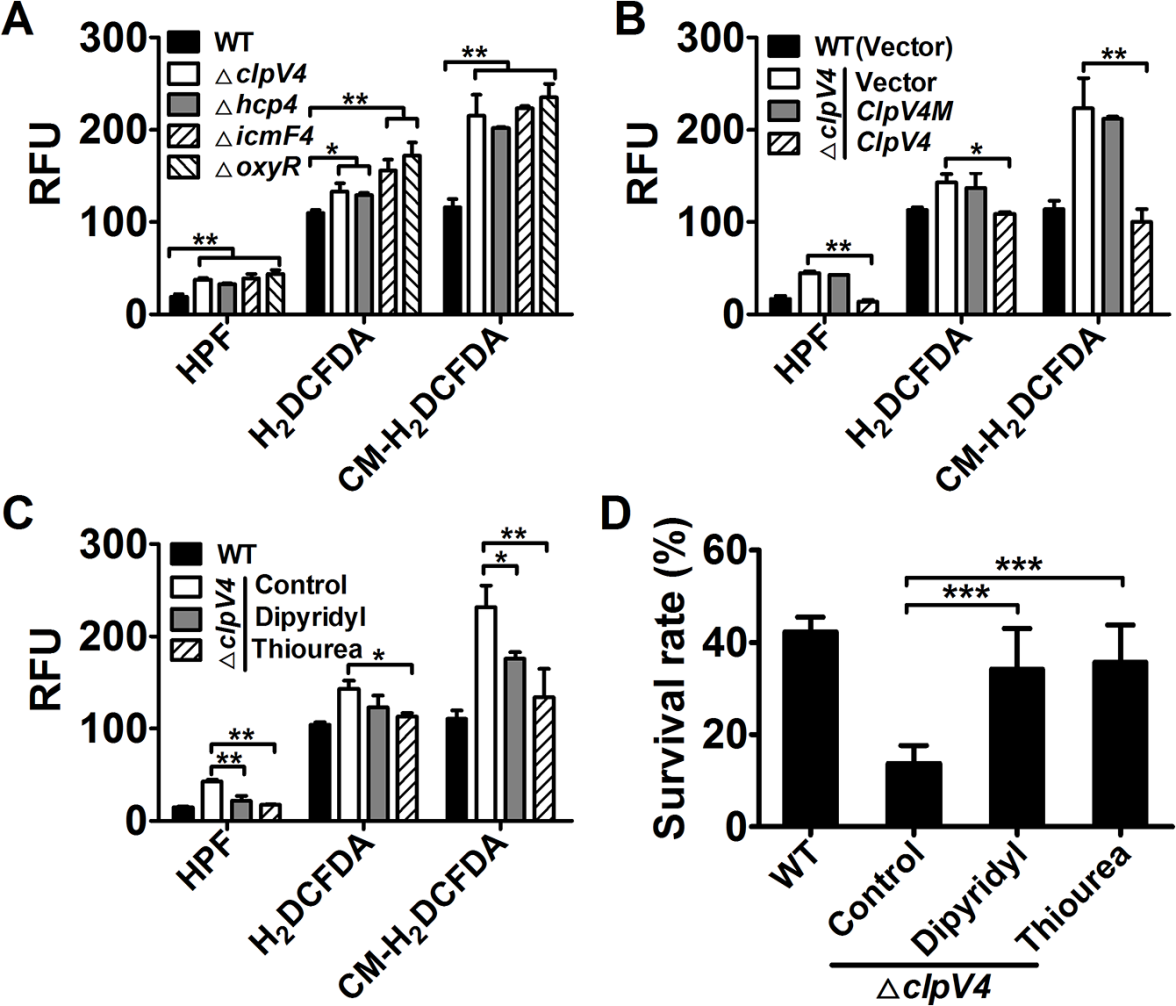
Deletion of T6SS-4 led to accumulation of intracellular ROS in *Yptb* under oxidative conditions. **A.** Oxidative stress induced the generation of intracellular ROS in T6SS-4 mutants. Intracellular ROS in mid-exponential phase bacteria exposed to H_2_O_2_ were stained with HPF, CM-H2DCFDA, or H2DCFDA dye; fluorescence signals were measured using a SpectraMax M2 Plate Reader (Molecular Devices) with excitation/emission wavelengths of 490/515 nm (HPF), 495/520 nm (CM-H2DCFDA and H2DCFDA). **B**. A functional T6SS-4 is required to eliminate cellular ROS. Note the inability of the *clpV4M* defective in ATPase activity to complement the mutation. **C**. Reduction of cellular ROS in the mutants by 2,2′-dipyridyl or thiourea. The compound was added to the bacterial cells subjected to oxidative stress challenge and the levels of ROS were measured. **D**. ROS mitigation agents rescued the sensitivity of T6SS-4 mutants to H_2_O_2_. 1 mM 2,2′-dipyridyl or 150 mM thiourea was added to bacterial cells challenged by oxidative stress and their survival rates were determined. In each case, higher levels of cellular ROS were indicated by higher fluorescence intensity. Data shown were the average of three independent experiments; error bars indicate SD from three independent experiments. ***, *p*<0.001; **, *p*<0.01; *, *p*<0.05.

Oxidative stress induces the production of deleterious reactive oxygen species (ROS), including the highly destructive hydroxyl radicals (HRs), which were generated via Fenton chemistry [16,17]. We thus used three fluorescent dyes with ranging specificity, to independently measure intracellular ROS levels in relevant *Yptb* strains challenged with H_2_O_2_. Among the three fluorescent dyes used, 2′,7′-dichlorodihydrofluorescein diacetate (H2DCFDA) detects H_2_O_2_ and ROO•, 3′-(*p*-hydroxyphenyl) fluorescein (HPF) detects HRs, and 5-(and-6)-chloromethyl-2′,7′-dichlorodihydrofluorescein diacetate, acetylester (CM-H2DCFDA) detects H_2_O_2_, ROO• and HRs [18–20]. Although the absolute units of the fluorescence signal varied, mutants lacking *oxyR* or essential components of T6SS-4 contained significantly higher amounts of ROS, especially HRs than wild-type bacteria in assays using each of the three ROS reporter dyes (Fig 3A). The ROS-induced fluorescence signals were specific because no signal was detected in control samples treated with H_2_O_2_ but without the dyes or with dyes but not treated with H_2_O_2_ (S2 Fig).

Expression of the corresponding genes eliminated the HRs accumulated in the mutants. For example, the level of HRs in the *ΔclpV4* mutant was almost completely eliminated by complementation with the wild-type gene but not by the *clpV4* mutant (*clpV4M*) defective in ATPase activity (Fig 3B). Consistently, treatment with chemical HRs mitigation agents 2,2′-dipyridyl or thiourea [21,22] reduced intracellular HRs levels induced by H_2_O_2_ in mutant bacteria (Fig 3C), further validating the notion that HRs accumulation contributes to bacterial death. Furthermore, when added into bacterial cultures challenged by oxidative stress, each of these two chemicals was able to increase the survival rates of T6SS-mutants to levels comparable to those of wild-type bacteria (Fig 3D), further validating the notion that HRs accumulation contributes to bacterial death. Together, these results indicate that T6SS-4 is critical in neutralizing HRs accumulated in *Yptb* under oxidative stress conditions.

### Intracellular accumulation of zinc ions under oxidative condition required T6SS-4

**Fig 4:**
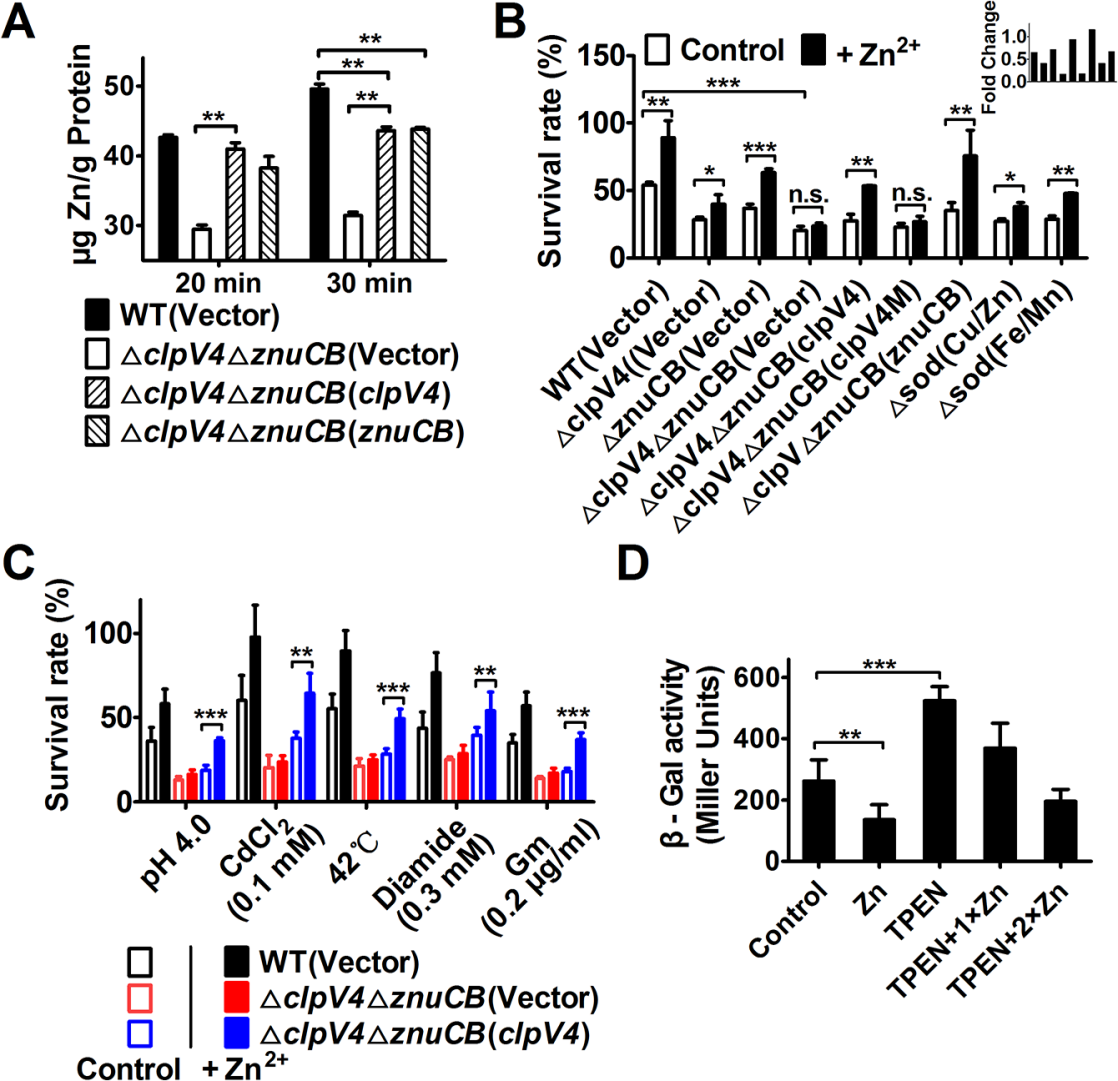
T6SS-4 is important for the accumulation of intracellular Zn^2+^ under oxidative stress conditions. **A.** Zn^**2+**^ uptake by relevant *Yptb* strains. Mid-exponential phase of *Yptb* strains were exposed to 1.5 mM H_2_O_2_ for 20 or 30 min in PBS containing 1 μM ZnCl_2_. Zn^**2+**^ associated with bacterial cells was measured by inductively coupled plasmon resonance atomic absorption spectrometry (ICP-MS). **B**. The alleviation of the sensitivity of *Yptb* mutants to H_2_O_2_ by exogenous Zn^**2+**^ required T6SS-4. *znuCB*, the canonical Zn^**2+**^ transporter; note that *clpV4M*, a mutant of *clpV4* defective in ATPase activity failed to complement the mutation. **C**. T6SS-4 is required for maximal bacterial survival in stress created by distinct agents. Mid-exponential phase bacteria were exposed to indicated agents or treatment for 1 hour (42°C for 30 min) and their survival was determined. **D**. T6SS-4 expression is induced by low zinc conditions. Cells of relevant *Yptb* strains harboring *T6SS-4p*::*lacZ* were grown in YLB medium with 100 μΜ Zn^**2+**^, 100 μΜ TPEN, 100 μΜ TPEN together with 100 μΜ Zn^**2+**^ (TPEN+1×Zn), or 100 μΜ TPEN together with 200 μΜ Zn^**2+**^ (TPEN+2×Zn), and the expression of the reporter was measured. Data shown were the average of three independent experiments; error bars indicate SD from three independent experiments. ***, *p*<0.001; **, *p*<0.01; *, p<0.05; n.s., not significant.

Metal ion homeostasis regulates cellular level of HRs [23]. For example, the manganese transporter MntABC and the zinc uptake system ZosA contribute to oxidative stress resistance in bacteria by mitigating HRs [24,25]. To test the hypothesis that T6SS-4 is involved in metal ion homeostasis, we measured the total metal contents in bacteria challenged with H_2_O_2_ using inductively coupled plasmon resonance atomic absorption spectrometry (ICP-MS). Our results revealed that deletion of T6SS-4 in the *ΔznuCB* background significantly lowered intracellular Zn^2+^ levels and that the expression of *znuCB* or *clpV4* partially restored such defects (Fig 4A). In contrast, the accumulation of Mn^2+^ was not affected in these mutants (S3 Fig). Such defects clearly did not result from potentially lower live bacterial cells because the 20 min treatment did not detectably affect bacterial viability (S4 Fig). Thus, T6SS-4 likely is involved in Zn^2+^ uptake. Consistent with this notion, exogenous Zn^2+^ was able to restore the growth of *Yptb* in H_2_O_2_ but only in the presence of a functional T6SS-4 or ZnuABC transporter (Fig 4B). Bacterial sensitivity to oxidative stress by mutants lacking both the canonical Zn transporter ZnuCB and T6SS-4 cannot be rescued by exogenous Zn^2+^ (Fig 4B). As expected, mutants lacking SOD(Cu/Zn) or SOD(Fe/Mn) are more sensitive to oxidative stress and such sensitivity can be partially rescued by exogenous Zn ions (Fig 4B). However, the sensitivity of Δ*znuCB*Δ*clpV4*(Vector) and Δ*znuCB*Δ*clpV4*(*clpV4M*) cannot be rescued by exogenous Zn ions (Fig 4B). In contrast, the sensitivity of strains Δ*znuCB*Δ*clpV4*(*znuCB*) and Δ*znuCB*Δ*clpV4*(*clpV4*) can be rescued by exogenous Zn ions (Fig 4B), further suggesting that T6SS-4 functions similarly to *ZnuCB* in zinc uptake.

The fact that the T6SS-4 participates in zinc acquisition predicts that the vegetative growth rate of the Δ*clpV4*Δ*znuCB* mutant will be affected by Zn^2+^ starvation under oxidative conditions. This prediction was confirmed by comparing the growth of the wild-type and the Δ*clpV4*Δ*znuCB* mutant in the presence of the Zn^2+^ chelator TPEN (N,N,N′,N′-tetrakis(2-pyridylmethyl) ethylenediamine) under H_2_O_2_ stress (S5 Fig). Whereas the growth of all tested strains is nearly identical in YLB medium (S5A Fig), the growth of the Δ*clpV4*Δ*znuCB* mutant is severely impaired compared to the wild-type in the presence of TPEN under H_2_O_2_ stress. The expression of either *znuCB* or *clpV4* from a complementation plasmid almost completely rescued the sensitivity of the Δ*clpV4*Δ*znuCB* mutant to TPEN (S5C Fig). Moreover, the growth defect of the mutant was completely rescued by the addition of excessive Zn^2+^, further supporting the role for T6SS-4 in Zn^2+^ acquisition (S5D and S5E Fig).

Because the production of HRs contributes to the cellular toxicity under diverse stress conditions [20,26], we reasoned that if T6SS-4 functions in Zn^2+^ uptake, it should be required for maximal bacterial survival under these stresses. Indeed, in the *ΔznuCB* mutant background, T6SS-4 is required for the resistance to diverse stress conditions such as low pH, high temperature, heavy metal, diamide and antibiotic (gentamicin) (Fig 4C). Taken together, these results point to a role of T6SS-4 in importing Zn^2+^ from the environment under diverse stress conditions.

The observation that TSSS-4 is involved in Zn^2+^ uptake points to the notion that the expression of this transporter should be responsive to low Zn^2+^ conditions. Indeed, the addition of exogenous Zn^2+^ repressed the expression of T6SS-4 in wild-type *Yptb* (Fig 4D). Chelating Zn^2+^ from the medium by TPEN led to robust expression from the promoter, and such induction can be repressed by exogenous zinc ions (Fig 4D). Thus, the expression of T6SS-4 is responsive to the levels of Zn^2+^ in the environment, which is consistent with its role in acquiring this metal ion from the extracellular milieu.

### T6SS-4 translocates a zinc-binding protein substrate

Zinc transport by T6SS-4 can be achieved by direct ion translocation via the secretion channel or by a Zn^2+^-binding carrier protein translocated by the secretion system. We distinguished between these two possibilities by analyzing predicted proteins adjacent to structural components of T6SS-4 for putative Zn^2+^-binding motifs with HHpred [27]. Such analyses revealed that Ypk_3549, a 117-residue protein encoded by a gene located at the end of the T6SS-4 gene cluster, contains a putative zinc finger motif (S6 Fig). No putative promoter can be identified upstream of *ypk_3549*, suggesting that this gene is part of the T6SS-4 operon. Indeed, similar to structural components of T6SS-4, the expression of *ypk_3549* is activated by OxyR under oxidative conditions (S7 Fig). BLAST analysis revealed that homologs of this gene are present in the genomes of *Yersinia pestis*, *Serratia marcescens* and possibly *Burkholderia oklahomensis*. Because Ypk_3549 is a putative substrate of T6SS-4 that may bind Zn^2+^, we designated it YezP (*Y* *ersinia* extracellular zinc-binding protein).

**Fig 5:**
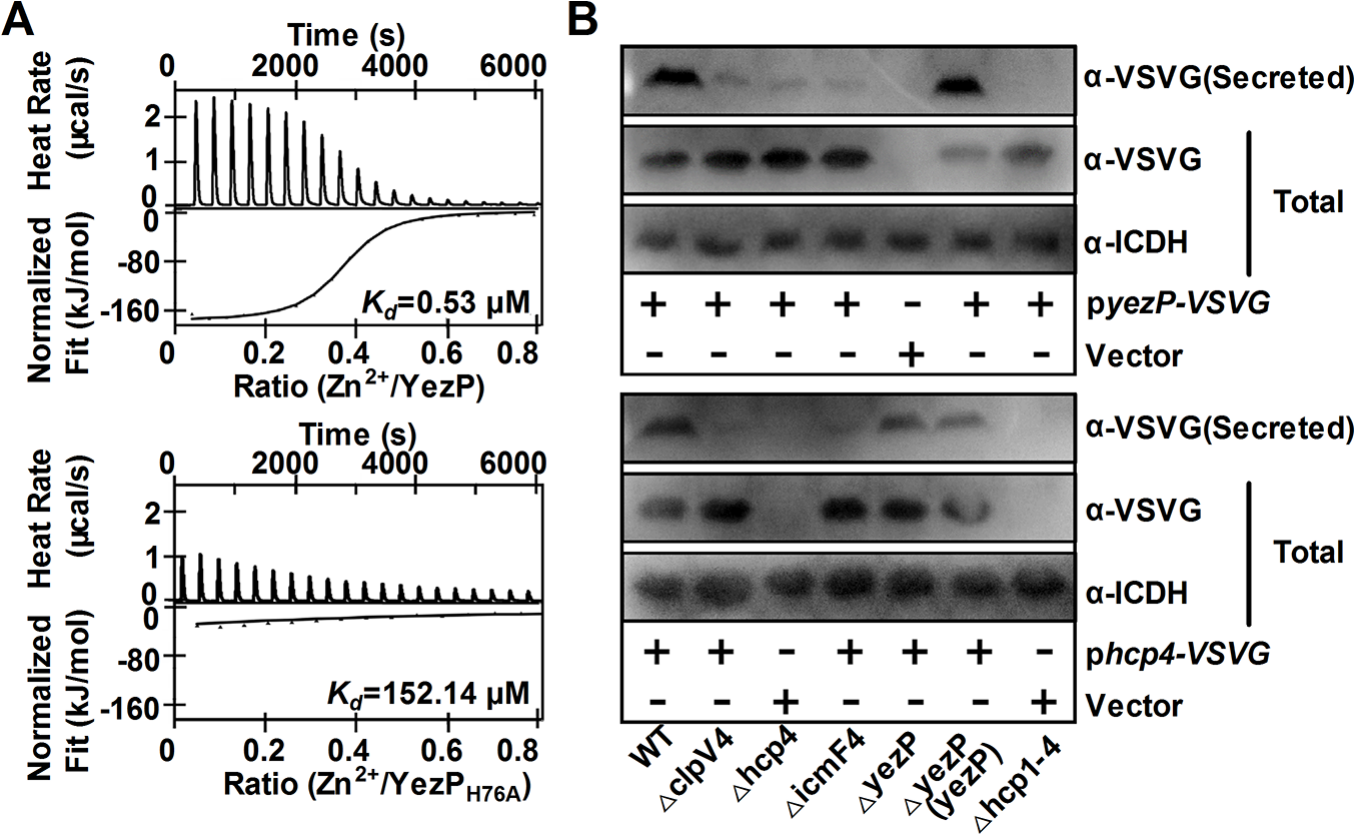
T6SS-4 translocates a Zn^2+^-binding protein. **A.** The binding of zinc ions by YezP. Zn^**2+**^-free YezP (upper) or YezP_H76A_ (lower) was used to evaluate zinc-binding activity by isothermal titration calorimetry (ITC). Data were analyzed with the NanoAnalyze software (TA Instruments). **B**. YezP is a secretion substrate of T6SS-4. Proteins in culture supernatant of relevant *Yptb* strains expressing YezP-VSVG were probed for VSVG (upper) or Hcp4-VSVG (lower) by immunoblotting. For the pellet fraction, the isocitrate dehydrogenase (ICDH) was detected as loading controls. Similar results were obtained in three independent experiments, and data shown are from one representative experiment done in triplicate.

Analysis with isothermal titration calorimetry [28] revealed that YezP bound Zn^2+^ with a *K*_*d*_ of 0.53 μM (Fig 5A upper panel). Importantly, mutation of residue histidine-76 predicted to participate in the formation of the zinc finger reduced its affinity to Zn^2+^ for more than 150-fold (*K*_*d*_ = 152.14 μM) (Fig 5A lower panel). Unexpectedly, the YezP_H76A_ mutant still bound to Zn^2+^, although at a markedly lower affinity. Similar Zn^2+^-binding activity of YezP and YezP_H76A_ was detected when the interaction was measured with 4-(2-pyridylazo) resorcinol (PAR) [29] (S8 Fig). However, this protein did not detectably bind to iron ions (S9 Fig), which differs from the zincophore yersiniabactin from *Yersinia* spp. capable of binding both zinc and iron ions [30]. The residual Zn^2+^ binding activity of YezP_H76A_ may result from a second noncannonical zinc-binding motif in the protein. Consistent with its zinc-binding activity and OxyR-dependent expression, YezP is required for Zn^2+^ accumulation in the cells in the *ΔznuCB* background (S10 Fig). Similarly, in line with the fact that low extracellular Zn^2+^ concentrations induced its expression, higher levels of YezP were detected in bacterial culture supernatant when Zn^2+^ was sequestered by the chelator TPEN (S11 Fig).

The above results suggest that T6SS-4 is involved in either the secretion or import of YezP. To distinguish between these two models, we expressed YezP-VSVG in relevant *Yptb* strains and examined its secretion. Significant amounts of YezP-VSVG can be readily detected in culture supernatant of wild-type bacteria (Fig 5B upper panel). Mutations in T6SS-4 structural genes almost completely abrogated the secretion of YezP; the residual secretion was completely abolished in a mutant lacking all 4 T6SSs in *Yptb* (Fig 5B upper panel). Furthermore, deletion of *yezP* did not affect the secretion of Hcp4 (Fig 5B lower panel), indicating that this protein was not involved in substrate secretion by T6SS-4. Interestingly, akin to YezP, the secretion of Hcp4 was completely abolished only in a mutant lacking all 4 T6SS of *Yptb* (Fig 5B lower panel), indicating the existence of limited substrate cross recognition among these transporters. These results establish that YezP is a substrate secreted by T6SS-4.

**Fig 6:**
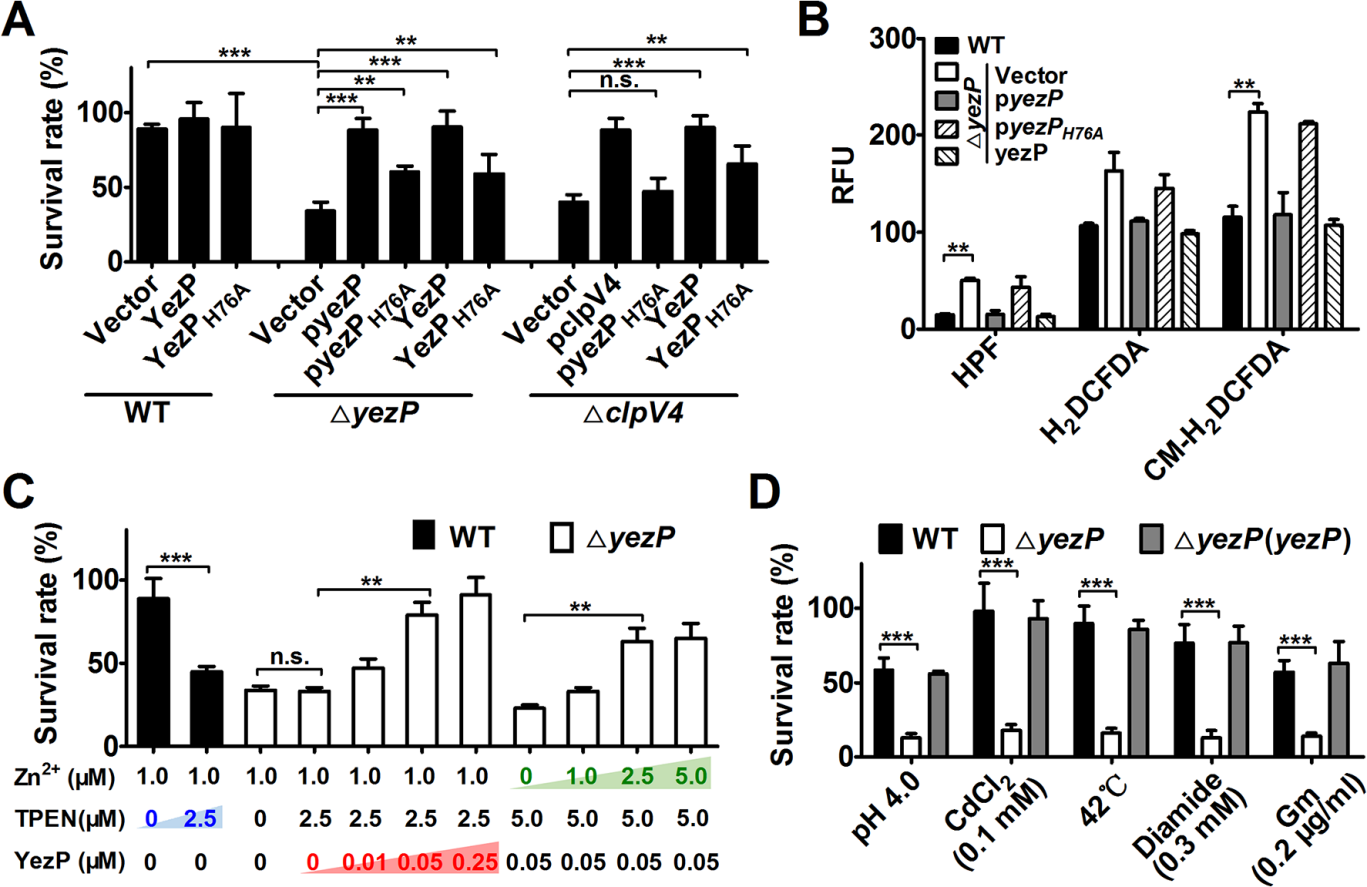
The activity of YezP in *Yptb* resistance to stresses. **A.** The rescue of the *yezP* mutant or a T6SS-4 mutant by recombinant YezP. 0.05 μM recombinant YezP or YezP_H76A_ was added to bacterial survival experiments before viability assessment. Mutants complemented with the corresponding gene were used as controls. Note the partial activity of YezP_H76A_. **B**. Deletion of *yezP* led to accumulation of intracellular ROS. Analysis of the mutants was performed as described in Fig 3 with the indicated fluorescence dyes. **C**. Recombinant YezP rescued the inhibition effects of a zinc chelator. TPEN and the indicated amounts of YezP or Zn^**2+**^ were incubated with bacterial cells prior to survival determination. **D**. *yezP* is required for the resistance to distinct cellular insults by *Yptb*. Bacteria were subjected to treatment with the indicated agents or conditions before determining bacterial survival rates. Gm, gentamicin. Data shown were the average of three independent experiments; error bars indicate SD from three independent experiments. ***, *p*<0.001; **, *p*<0.01; n.s., not significant.

That YezP is a zinc-binding substrate of T6SS-4 suggests that it is required for maximal bacterial survival under oxidative challenge. Indeed, the *ΔyezP* strain exhibited sensitivity to H_2_O_2_ at levels similar to those of T6SS-4 mutants and such sensitivity can be fully complemented by wild-type, and partially by the H76A mutant which still retains residual Zn^2+^ binding activity (Fig 6A). We next determined whether recombinant YezP restored the ability of relevant *Yptb* mutants to survive oxidative challenge. Inclusion of recombinant YezP in cultures of the *ΔyezP* strain fully restored its resistance to H_2_O_2_ (Fig 6A). More importantly, recombinant YezP protein also protected the T6SS-4 mutant *ΔclpV4* from toxicity imposed by H_2_O_2_ (Fig 6A), indicating that after T6SS-4-mediated translocation, zinc uptake by YezP occurs independently of the secretion system. Consistent with its partial Zn^2+^-binding activity, YezP_H76A_ still detectably conferred resistance to oxidative stress in mutants defective in T6SS-4 or its coding gene (Fig 6A). In agreement with its role in Zn^2+^ acquisition to neutralize HRs, deletion of *yezP* resulted in accumulation of these harmful agents in bacterial cells to levels similar to those observed in T6SS-4 mutants (Fig 6B). Such accumulation can be eliminated by either expression of *yezP* from a plasmid or by recombinant YezP protein (Fig 6B).

We further determined the importance of Zn^2+^ sequestration by recombinant YezP by adding the zinc chelator TPEN to the protein solution used for complementation. The inhibitory effects of TPEN can be neutralized by recombinant YezP in a dose-dependent manner (Fig 6C). Although the addition of 0.05 μΜ YezP protein increased the survival rate of the *ΔyezP* mutant treated with 2.5 μΜ TPEN, it did not increase the survival rate of the same mutant in the presence of 5 μΜ TPEN when the concentration of Zn^2+^ was 1.0 μM. Instead, the inhibition by 5 μΜ TPEN in the presence of 0.05 μΜ YezP can be significantly reversed by the addition of exogenous zinc (Fig 6C). Similar to T6SS-4 mutants, the *ΔyezP* mutant was sensitive to agents such as low pH, heavy metal, high temperature, diamide and antibiotic (gentamicin) that induce HRs production [17] (Fig 6D), further indicating that zinc transport by T6SS-4 functions to combat cellular stress induced by a wide spectrum of environmental cues.

### *Yptb* mutants lacking T6SS-4 or *yezP* are defective in virulence in mice

**Fig 7:**
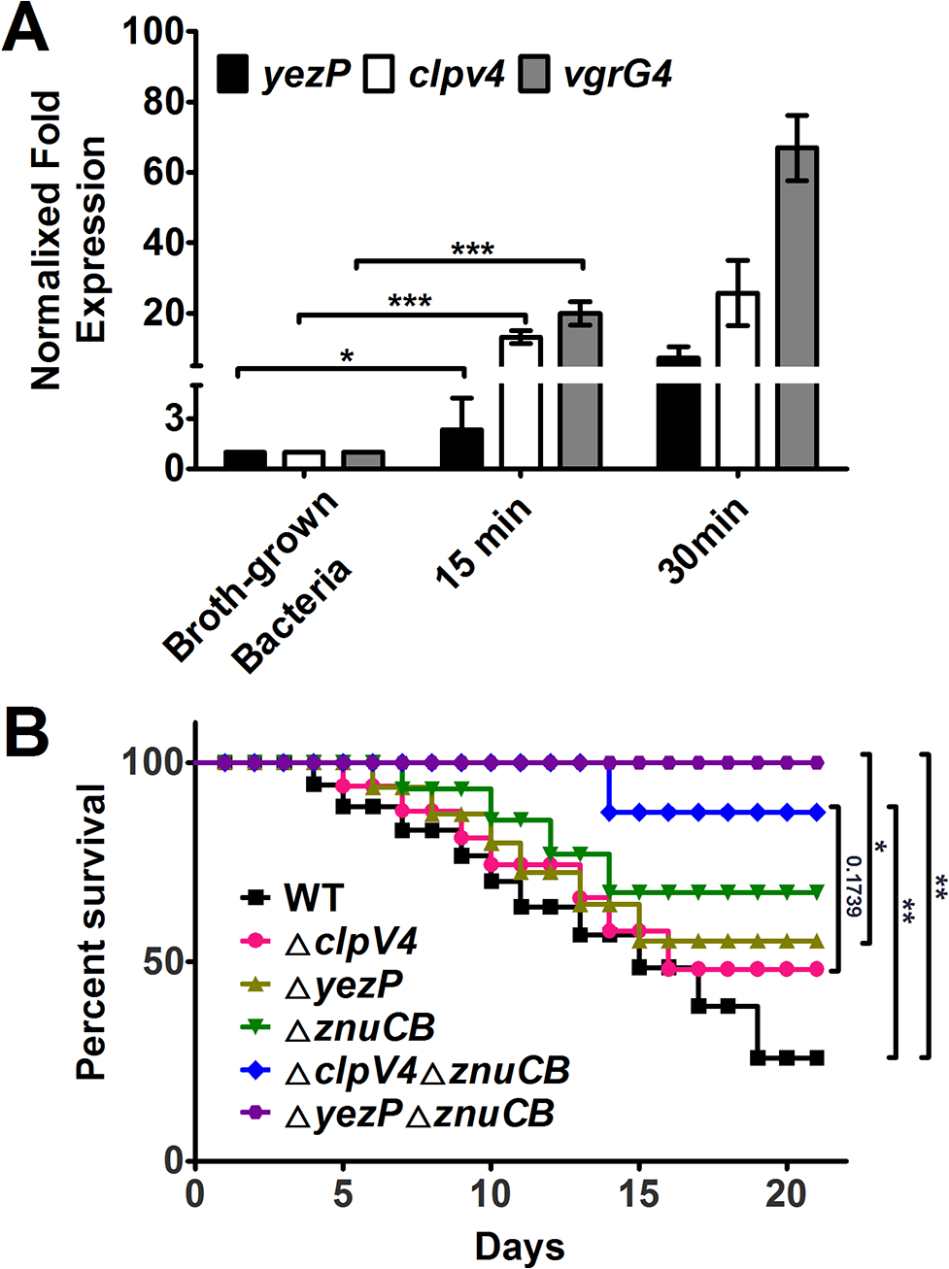
The expression of T6SS-4 is induced in macrophages and *Yptb* mutants lacking T6SS-4 or its substrate YezP are defective in virulence against mice. **A.** The expression of *yezP* and T6SS-4 is induced in macrophages. Wild-type *Yptb* was used to infect bone marrow-derived macrophages at an MOI of 10 for 15 or 30 min, and the expression of *yezP*, *clpV4* and *vgrG4* was measured by qRT-PCR. Bacteria grown in YLB were used as controls. Data shown were the average of three independent experiments; error bars indicate SD from three independent experiments. ***, *p*<0.001; *, *p*<0.05. Statistic analyses were performed by Student’s *t*-test. **B.** Bacterial strains grown in YLB were washed twice in sterilized PBS and used for orogastric infection of 6–8 weeks old female C57BL/6 mice using a ball-tipped feeding needle. For survival assays 3×10^**9**^ bacteria of each strain were applied to different groups of mice (n = 10/strain), and the survival rate of the mice was determined by monitoring the survival daily for 3 weeks. Similar results were obtained in three independent experiments, and data shown are from one representative experiment done in triplicate. **, *p*<0.01; *, *p*<0.05. Statistic analyses were performed by Log-Rank test.

The host immune system imposes significant stress to a pathogen. *Yptb* is an enteric pathogen with a tropism for lymphoid tissue; it also hijacks macrophages as Trojan horses for dissemination and subsequent systemic infection [31]. Oxidative burst is an important microbial killing mechanism in phagocytes. We thus tested the induction of T6SS-4 after the bacterium being phagocytosed by primary macrophages. Wild-type *Yptb* was used to infect primary macrophages from C57BL/6 mice for different durations and the expression of *yezP*, *clpV4* and *vgrG4* was measured by qRT-PCR. Compared to broth grown bacteria, the expression of *clpV4* and *vgrG4* was induced for about 13–20 folds 15 min after infection, and 25–60 folds of induction was detected at 30 min post infection (Fig 7A). Although at lower rates, the expression of *yezP* was also significantly induced in phagocytosed bacteria (Fig 7A).

The induction of T6SS-4 in primary macrophages suggests that this transporter may be important for the virulence of *Yptb* in animal infection. We thus examined this hypothesis by orogastrically inoculating relevant bacterial strains into C57BL/6 mice. Wild-type bacteria caused more than 50% lethality within two weeks of inoculation. On the other hand, consistent with the stress sensitivity phenotypes, mice infected with mutants lacking T6SS-4 or *yezP* survived better at similar rates (Fig 7B). Similarly, the mutant lacking the classical zinc transporter *znuCB* that plays important roles in competition with vertebrate host for Zn^2+^ is less virulent to mice [30]. Notably, mutations of *znuCB* together with T6SS-4 or *yezP* resulted in mutants that almost completely lost the virulence against mice (Fig 7B), further implying the importance of T6SS-4 in the resistance to host nutritional immunity. Thus, zinc transportation by T6SS-4 plays an important role in the interactions of *Yptb* with mammalian hosts.

## Discussion

The best-studied function of T6SSs is their role in the killing of competing species in specific niches using bacteriolytic effectors [2]. Previous studies have suggested roles of T6SSs in nonbiological challenges such as stress resistance [13,14], but the underlying mechanisms remain largely unknown. In this report, we found that the T6SS-4 from *Y*. *pseudotuberculosis* functions to combat multiple adverse stresses and host nutritional immunity by translocating a zinc-binding effector.

Bacterial cells need to deal with insults of distinct origins in different phases of their life cycle or in different environmental niches. The production of detrimental HRs is emerging as a potentially important consequence of diverse environmental challenges [17,20,26]. In addition to their roles as cofactors in many essential enzymes, transition ions such as Zn^2+^ and Mn^2+^ are capable of mitigating HRs to reduce their damage [24,32]. Consistent with the notion that T6SS-4 functions to combat oxidative stress induced by diverse cues, its expression is also induced by high osmolality and low pH conditions via the osmotic/acid stress regulator OmpR [13,14], which is consistent with its role in the resistance to a broad range of adverse stresses. Similarly, although the mechanism has not yet been well studied, a T6SS in *Vibrio anguillarum* is regulated by the general stress response regulator RpoS and is involved in its resistance to hydrogen peroxide, ethanol and low pH [12]. The response of T6SS-4 to distinctly different signals via multiple regulatory proteins [13,14] and the role of Zn^2+^ in HRs mitigation provide a molecular explanation for “cross-protection”, a phenomenon in which bacterial cells subjected to one stressful condition often became resistant to stress created by distinctly different insults [33].

**Fig 8:**
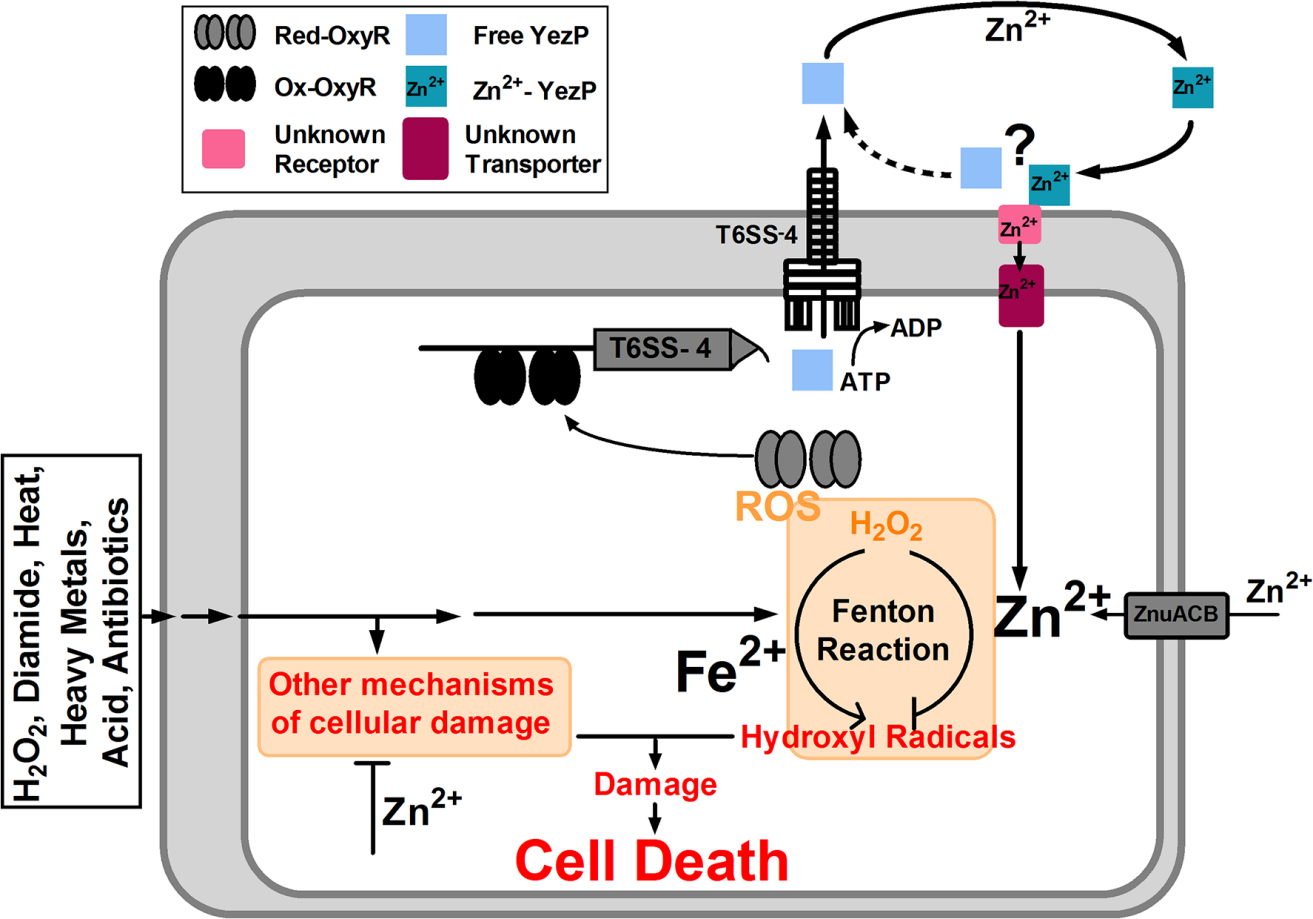
Model of T6SS-4-facilitated Zn^2+^ transportation and oxidative resistance in *Yptb*. OxyR activated by oxidative signals binds to the operator in the promoter of T6SS-4 to activate the expression of the system and its substrate YezP, leading to the production and assembly of the system, which translocates YezP into the extracellular milieu. YezP form a complex with Zn^**2+**^ to deliver the ions into the cell via a yet unknown mechanism. Intracellular Zn^**2+**^ mitigates the hydroxyl radicals or potentially other cell damaging processes to make the cells resistant to diverse environmental challenges.

Metal ions can be transported into bacterial cells by specific ion transporter [34] or by chelators, such as siderophores, which are high-affinity iron-binding molecules [35]. Our discovery of T6SS in bacterial ion acquisition significantly expanded the function of specialized protein secretion system. Due to their multiple components, the expression and assembly of T6SSs presumably will consume more energy. As a result, this system may only be activated when metal ions imported by the more classical transporters are not sufficient. Alternatively, the expression or activity of the classical metal ion transporters may be inhibited under certain environmental conditions. We proposed a model that under normal conditions, the ZnuCB transporter fulfills the need of Zn^2+^ of the cells, and the T6SS-4 in *Yptb* contributes to this process as a Zn^2+^ scavenger when the bacterium encounters stress conditions that lead to the production of HRs and potentially other cell damaging mechanisms by oxidative or other adverse conditions capable of activating OxyR (Fig 8). The establishment of a role of T6SS in dealing with non-biological challenges may provide an explanation to the wide spread of these protein secretion systems. It will be of great interest to determine whether bacteria not normally associated with a eukaryotic host employ T6SS to facilitate their survival in unfavorable environmental conditions.

For pathogenic bacteria, one benefit of the metal ion acquisition activity of T6SS is to compete for Zn^2+^ within the host, or other essential metal ions to fight against nutritional immunity [36]. Given the essential role of Zn^2+^ in bacterial physiology, it is not surprising that the ability of T6SS-4 in acquiring Zn^2+^ might offer *Yptb* an advantage in pathogenesis because Zn^2+^ in mammalian hosts is strictly sequestered by a defense mechanism termed nutritional immunity [36]. Indeed, the classical zinc transporter ZnuCB was found to play pivotal roles in competition against vertebrate host for Zn^2+^ [36–38]. Accordingly, deletion of *znuCB* attenuated the pathogenicity of important pathogens such as *Acinetobacter baumannii* [38], *Brucella abortus* [39] and *Campylobacter jejuni* [40]. Consistently, we found *Yptb* T6SS-4 mutants are attenuated in virulence against mice. Notably, mutations of T6SS-4 or *yezP* together with *znuCB* resulted in mutants that are almost completely avirulent in a mouse infection model, indicating the importance of T6SS-4 in the resistance to host nutritional immunity. These results may also explain the observation that mutations in *znuCB* did not affect the virulence of *Y*. *pestis* [41]. Interestingly, the siderophore yersiniabactin has recently been shown to participate in Zn^2+^ acquisition in *Y*. *pestis* [42]. Furthermore, mutants lacking both the ZnuABC system and yersiniabactin are defective in virulence [42]. The fact that the *ybt* locus for yersiniabactin biosynthesis are absent in the *Yptb* strain YpIII used in our current study (http://www.ncbi.nlm.nih.gov/nuccore/169748796) may explain the strong phenotypes of mutants defective in both T6SS-4 and ZnuCB. It will be interesting to determine whether *Y*. *pestis* mutants lacking all three known Zn^2+^ acquisition systems display further reduction in virulence.

Zn^2+^ transported by T6SS-4 of *Y*. *pestis* may compensate the effects caused by the loss of the canonical transporter. Lethal systemic infection by *Yptb* has multiple phases, including initial survival in the gastrointestinal track, invasion through M-cells into the Peyer’s patches and the subsequent trafficking to the deep tissue via macrophages [43]. The observed loss of virulence can result from impairment in the competitiveness against the microflora prior to infection or by the inability to compete with Zn^2+^ sequestration mechanisms in host cells or a combination of both. Hence, this finding provided a new perspective for revealing the mechanisms of T6SS in pathogenesis.

Zinc acquisition by secreted zinc-binding proteins seems to be a mechanism shared by taxonomically diverse microorganisms. Secreted Zn^2+^-chelating compounds (zincophores) analogous to siderophores have been identified in pathogenic bacteria such as *Pseudomonas aeruginosa* [44] and *Y*. *pestis* [42]. Recently, the Zn^2+^-binding protein Pra1 important for zinc acquisition from hosts by the fungal pathogen *Candida albicans* was identified [45]. Similar to Pra1, YezP appears to contain multiple Zn^2+^-binding motifs (Fig 5). In *Mycobacterium tuberculosis*, the type VII secretion system ESX-3 is necessary for optimal growth in zinc-limited conditions, implying the involvement of a similar mechanism in zinc acquisition by this pathogen [46].

The affinity of YezP for Zn^2+^ (*K*_*d*_ = 0.53 μM) is considerably lower than that of canonical Zn importers such as ZnuA (<20 nM) [47] and ZinT (22 nM) [48], but it is comparable to or even higher than other Zn importers like YiiP (1000 nM) [49], the cation diffusion facilitator (CDF) (865 nM) [50] and the membrane zinc transporter (MTP1) (23 μM) [51]. Despite the relatively lower affinity for Zn^2+^, YezP is crucial for *Yptb* survival in stress conditions or for successful colonization of a mammalian host, particularly in the absence of the canonical Zn^2+^ transporter ZnuCB. Under our experimental conditions, even in the presence of strong chelators such as TPEN, recombinant YezP was able to provide Zn^2+^ sufficient for the cells to survive under oxidative stress (Fig 6). Future elucidation of the mechanism of the transfer of Zn^2+^ to the cell by YezP may explain how this protein functions in the presence of a chelator with much higher affinity for the ions. Similarly, the exact mechanism and timing of YezP’s contribution to the infection process needs further investigations.

The Zn^2+^-binding protein substrate YezP represented a novel type of T6SS effectors distinct from those extensively-studied bacteriolytic toxins or eukaryotic cell-targeting effectors. Our study suggests that T6SS-4 secretes a proteinaceous Zn^2+^ chelator as a strategy to acquire this nutrient, which implies the existence of a mechanism for subsequent acquisition of zinc from this protein by bacterial cells. Clearly, this process occurs independent of T6SS-4 as recombinant YezP was able to rescue the sensitivity of its mutant to oxidative stress. Evidently, the implication of ion transport by T6SS is broader than bacterial interactions with hosts or competing species. The ability to more effectively acquire nutrients is beneficial to the bacteria, pathogenic or environmental species, when they encounter challenges in specific niches. Future study aiming at the identification of the machinery for importing Zn^2+^ from YezP or the Zn^2+^/protein complex surely will lead to a better understanding of mechanism not only for the function of these widely distributed secretion systems, but also for metal ion uptake by bacteria.

## Materials and Methods

### Ethics statement

All mouse experimental procedures were performed in accordance with the Regulations for the Administration of Affairs Concerning Experimental Animals approved by the State Council of People’s Republic of China. The protocol was approved by the Animal Welfare and Research Ethics Committee of Northwest A&F University (protocol number: NWAFU 2014002).

### Bacterial strains and growth conditions

Bacterial strains and plasmids used in this study are listed in S1 Table. *Escherichia coli* were grown in LB with appropriate antibiotics at 37°C. *Y*. *pseudotuberculosis* (*Yptb*) strains were cultured in Yersinia-Luria-Bertani (YLB) broth (1% tryptone, 0.5% yeast extract, 0.5% NaCl) or M9 medium (Na_2_HPO_4_, 6g L^-1^; KH_2_PO_4_, 3g L^-1^; NaCl, 0.5g L^-1^; NH_4_Cl, 1g L^-1^; MgSO_4_, 1 mM; CaCl_2_, 0.1 mM; glucose 0.2%) at 26°C with appropriate antibiotics when necessary. The *Y*. *pseudotuberculosis* strain YPIII was the parent of all derivatives used in this study. In-frame deletions were generated by the method described previously [13]. Antibiotics were added at the following concentrations: nalidixic acid, 15 μg ml^-1^; ampicillin, 100 μg ml^-1^; kanamycin, 50 μg ml^-1^; tetracycline, 10 μg ml^-1^; chloramphenicol, 30 μg ml^-1^.

### Plasmid construction

Primers used in this study are listed in S2 Table. The *lacZ* fusion reporter vectors pDM4-*T6SS1-4p*::*lacZ* were made in our previous study [52]. To construct the *lacZ* fusion reporter vector pDM4-*katGp*::*lacZ*, primers *katGp*-*Sal*I-F/*KatGp*-*Xba*I-R were used to amplify the 585 bp *katG* promoter fragment from *Yptb* genomic DNA. The PCR product was digested with *Sal*I/*Xba*I and inserted into similarly digested pDM4-*T6SS-4p*::*lacZ* to produce pDM4-*katGp*::*lacZ*. To construct T6SS-4 promoter with mutations in the OxyR binding site, overlap PCR was performed to replace the consensus binding sites (60 bp) with identical amount of irrelevant base pairs. Briefly, to replace the OxyR binding site, primer pairs T6SS-4p-*Sal*I-F/T6SS-4pM-R and T6SS-4pM-F/T6SS-4p-*Xba*I-R were used to amplify the up-fragment and down-fragment of T6SS-4 promoter, respectively. Overlap PCR was carried out using the up-fragment and down-fragment as template and T6SS-4p-*Sal*I-F/T6SS-4p-*Xba*I-R as primer pair to obtain the DNA fragment T6SS-4pM. This fragment was further digested with *Sal*I and *Xba*I and inserted into similar digested pDM4-*T6SS-4p*::*lacZ* to construct pDM4-*T6SS-4pM*::*lacZ*.

The plasmid pDM4-*ΔoxyR* (*ypk_*4079) was used to construct the *ΔoxyR* in-frame deletion mutant of *Yptb*. A 918-bp and a 900-bp fragments flanking *oxyR* were amplified with primer pair *oxyR*-1F-*Bgl*II/*oxyR*-1R and *oxyR*-2F/*oxyR*-2R-*Sal*I, respectively. The upstream and downstream PCR fragments were ligated by overlap PCR. The resulting PCR products were digested with *Sal*I and *Bgl*II, and inserted into the *Sal*I/*Bgl*II site of pDM4 to produce pDM4-*ΔoxyR*. The knock-out plasmid pDM4-*ΔznuCB* (*ypk*_2141–2142), pDM4-*ΔicmF4* (*ypk*_3550), pDM4-*Δhcp1* (*ypk*_0385), pDM4-*Δhcp2* (*ypk*_0803), pDM4-*Δhcp3* (*ypk*_1481), pDM4-*ΔkatG* (*ypk*_3388), pDM4-*Δsod*(*Fe/Mn*) (*ypk*_1863), pDM4-*Δsod*(*Cu/Zn*) (*ypk*_3445) and pDM4-*ΔyezP* (*ypk*_3549) were constructed in similar manners by using primers list in S2 Table.

To complement the *oxyR* mutant, primers *oxyR*-F-*Sph*I/*oxyR*-R-*Sal*I were used to amplify the *oxyR* gene fragment from *Yptb* genomic DNA. The PCR product was digested with *Sph*I/*Sal*I and was inserted into similarly digested pKT100 to produce pKT100-*oxyR*. The complementary plasmid pKT100-*znuCB* and pKT100-*yezP* was constructed in similar manners using primers *znuCB*-F-*Sph*I/*znuCB*-R-*Sal*I and *3549*-F-*Sph*I/*3549*-R-*Sal*I, respectively. The complementary plasmid pKT100-*clpV4* and pKT100-*clpV4M* were made in our previous study [13].

Site-directed mutagenesis was carried out by overlap PCR to substitute the histidine residue at position 76 of YPK_3549 (YezP) into an alanine residue (YezP_H76A_). Briefly, DNA of mutant YezP_H76A_ was amplified by two rounds of PCR. Primer pairs *3549*-F-*Sph*I/*3549*-H76A-R and *3549*FH76A-F/*3549*-R-*Sal*I were used to amplify segments 1 and 2 respectively. The second round of PCR was carried out by using *3549*-F-*Sph*I/*3549*-R-*Sal*I as primer pair while fragment 1 and fragment 2 as templates to obtain the YezP_H76A_ fragment. The YezP_H76A_ DNA fragment was digested by *Sph*I/*Sal*I and cloned into similar digested pKT100 to produce pKT100-*yezP*_*H76A*_.

To express His_6_-tagged OxyR and Fur (ferric uptake regulator; *ypk*_2991), primers *oxyR*-F-*BamH*I/*oxyR*-R-*Sal*I and *fur*-F-*BamH*I/*fur*-R-*Sal*I were used to amplify *oxyR* and *fur* fragments from genomic DNA of *Yptb*. The PCR products of *oxyR* and *fur* were digested with *BamH*I/*Sal*I and inserted into the *BamH*I/*Sal*I sites of pET28a resulting in plasmids pET28a-*oxyR* and pET28a-*fur*. To express GST-tagged YezP, primers *3549*-F-*BamH*I and *3549*-R-*Sal*I were used to amplify the *yezP* gene from genomic DNA of *Yptb*. The PCR product of *yezP* was digested with *BamH*I/*Sal*I and inserted into the *BamH*I/*Sal*I sites of pGEX6P-1 resulting in plasmid pGEX6P-1-*yezP*. To construct the site-directed mutagenesis plasmid pGEX6P-1-*yezP*_*H76A*_, primers *3549*-F-*BamH*I and *3549*-R-*Sal*I were used to amplify the *yezP*_*H76A*_ fragment from pKT100-*yezP*_*H76A*_ plasmid DNA and was subcloned into similarly digested pGEX6P-1.

Plasmids pME6032-*yezP-VSVG* was constructed for protein secretion assay. Briefly, primers *3549*-F-*Eco*RI and *3549*taa-R-VSVG-*Bgl*II were used to amplify the *yezP* gene from *Yptb* genomic DNA. The PCR product of *yezP-VSVG* were digested with *Eco*RI/*Bgl*II and inserted into the *Eco*RI/*Bgl*II site of pME6032 to generate pME6032-*yezP-VSVG*. The plasmid pME6032-*hcp4-VSVG* was constructed in similar manners by using primers *hcp4*-F-*Eco*RI and *hcp4*taa-R-VSVG-*Bgl*II.

For complementation, complementary plasmids pKT100-*oxyR*, pKT100-*znuCB*, pKT100-*clpV4*, pKT100-*clpV4M*, pKT100-*yezP* and pKT100-*yezP*_*H76A*_ were introduced into respective mutants by electroporation. The integrity of the insert in all constructs was confirmed by DNA sequencing.

### Overexpression and purification of recombinant protein

To express and purify His_6_- and GST-tagged recombinant proteins, pET28a and pGEX6p-1 derivatives were transformed into *E*. *coli* BL21(DE3) and XL1-Blue competent cells, respectively. For protein production, bacteria were grown at 37°C in LB medium to an OD_600_ of 0.5, shifted to 22°C and then induced with 0.2–0.4 mM IPTG, and cultivated for an additional 12 h at 22°C. Harvested cells were disrupted by sonification and purified with the His•Bind Ni-NTA resin or GST•Bind Resin (Novagen, Madison, WI) according to manufacturer’s instructions. The GST tag was removed by incubation with PreScission Protease (GE healthcare) for 20 h at 4°C, and tag removed proteins were eluted from the GST•Bind Resin with PBS. The purity of the purified protein was verified as >95% homogeneity with SDS-PAGE analysis. Protein concentrations were determined by the Bradford assay [53].

### Protein secretion assay

Secretion assays for YezP (Ypk_3549) and Hcp4 were performed according to described methods [54]. All samples used for secretion assays in this study were taken at mid-exponential phase corresponding to an OD_600_ = 1.5–2.0. Briefly, strains were inoculated into 100 ml YLB broth and incubated with continuous shaking until OD_600_ reached 1.5–2.0 at 26°C. 2 ml culture was centrifuged and the cell pellet was resuspended in 100 μl SDS-sample buffer; this whole-cell lysate sample was defined as YezP_IN_. 90 ml of the culture was centrifuged, then the supernatant was filtered through a 0.22 μm filter (Millipore, MA, USA), and the proteins were extracted by filtration over a nitrocellulose filter (BA85) (Whatman, Germany) three times. The filter was soaked in 100 μl SDS sample buffer for 15 min at 65°C to recover the proteins present, and the sample was defined as YezP_OUT_. All samples were normalized to the OD_600_ of the culture and volume used in preparation. Secretion assays for Hcp4 was carried out by a similar procedure.

### Western blot analysis

Western blot analysis was performed as previously described [13]. Samples were resolved by SDS-PAGE and transferred onto PVDF membranes (Millipore). The membrane was blocked in 5% (w/v) nonfat milk for 4 h at room temperature, and incubated with primary antibodies at 4°C overnight: anti-Hcp4, 1:500; anti-VSVG (Santa Cruz biotechnology, USA), 1:500; anti-ICDH, 1:6000; anti-Pgi, 1: 2,000. The membrane was washed three times in TBST buffer (50 mM Tris, 150 mM NaCl, 0.05% Tween 20, pH 7.4), and incubated with 1:5,000 dilution of horseradish peroxidase conjugated secondary antibodies (Shanghai Genomics) for 1 h. Signals were detected using the ECL plus kit (GE Healthcare, Piscataway, NJ) following the manufacturer's specified protocol. The Hcp4, Pgi and ICDH antisera were made in our previous studies [13,55].

### Construction of chromosomal fusion reporter strains and β-galactosidase assays

The *lacZ* fusion reporter vectors pDM4-*T6SS-1p*::*lacZ*, pDM4-*T6SS-2p*::*lacZ*, pDM4-*T6SS-3p*::*lacZ*, pDM4-*T6SS-4p*::*lacZ* and pDM4-*katGp*::*lacZ* were transformed into *E*. *coli* S17-1λpir and mated with *Yptb* strains according to the procedure described previously [13]. The *lacZ* fusion reporter strains were grown in YLB broth and **β**-galactosidase activity was assayed with ONPG as the substrate [56]. The **β**-galactosidase results shown represent the mean of one representative assay performed in triplicate, and error bars represent standard deviation. Statistical analysis was carried out with Student’s *t*-test.

### DNase I footprinting assay

DNase I footprinting assays were performed according to [57] with minor modifications. The promoter region of T6SS-4 was PCR amplified with primers T6p4 footprinting-F/T6p4 footprinting-R and the fragment was cloned into the pMD-18T vector (TaKaRa), which was further used as the template for preparation of fluorescent FAM labeled probes with primers M13R(FAM-labeled) and M13F(-47). The FAM-labeled probes were purified by the Wizard SV Gel and PCR Clean-Up System (Promega) and quantified with NanoDrop 2000C (Thermo). For the DNase I footprinting assay, 400 ng probes were incubated with different amounts of His_6_-OxyR in a total volume of 40 μl in the same buffer. After incubation for 30 min at 30°C, 10 μl solution containing about 0.010 unit DNase I (Promega) and 100 nmol freshly prepared CaCl_2_ was added and further incubate for 1 min at 25°C. The reaction was stopped by adding 140 μl DNase I stop solution (200 mM unbuffered sodium acetate, 30 mM EDTA and 0.15% SDS). Samples were then extracted with phenol/chloroform, precipitated with ethanol and the pellets were dissolved in 35 μl MiniQ water. The preparation of the DNA ladder, electrophoresis and data analysis were the same as described before [57], except that the GeneScan-LIZ500 size standard (Applied Biosystems) was used.

### Electrophoretic mobility shift assay (EMSA)

Electrophoretic mobility shift assay was performed using biotin 5′-end labeled promoter probes. Bio-T6SS-4p and Bio-T6SS-4pM, amplified from pDM4-*T6SS-4p*::*lacZ* and pDM4-*T6SS-4pM*::*lacZ*, respectively, with primers *T6p*-*oxyR*-F-5′biotin/*T6p-oxyR*-R-5′biotin. The unlabeled T6SS-4p competitor DNA was amplified from pDM4-*T6SS-4p*::*lacZ* with primers *T6p*-*oxyR*-F/*T6p*-*oxyR*-R. All PCR fragments were purified by EasyPure Quick Gel Extraction Kit (TransGen Biotech, Beijing, China). Each 20-μl EMSA reaction solutions were prepared by adding the following components according to the manufacturer’s protocol (LightShift Chemiluminescent EMSA kit; Thermo Fisher Scientific): 1×binding buffer, 50 ng poly (dI-dC), 2.5% glycerol, 0.05% NP-40, 5 mM MgCl_2_, 20 fmol Biotin-DNA, 4 pmol unlabeled DNA as competitor and different concentrations of proteins. Reaction solutions were incubated for 20 min at room temperature. The protein-probes mixture was separated in a 6% polyacrylamide native gel and transferred to a Biodyne B Nylon membrane (Thermo Fisher Scientific). Migration of biotin-labeled probes was detected by streptavidin-horseradish peroxidase conjugates that bind to biotin and chemiluminescent substrate according to the manufacturer’s protocol.

### Quantitative real-time PCR

Bacteria were harvested during the mid-exponential phase and RNA was extracted using the RNAprep Pure Cell/Bacteria Kit and treated with RNase-free DNase (TIANGEN, Beijing, China). The purity and concentration of the RNA were determined by gel electrophoresis and spectrophotometer (NanoDrop, Thermo Scientific). First-strand cDNA was reverse transcribed from 1 μg of total RNA with the TransScript First-Strand cDNA Synthesis SuperMix (TransGen Biotech, Beijing, China). Quantitative real-time PCR (qRT-PCR) was performed in CFX96 Real-Time PCR Detection System (Bio-Rad, USA) with TransStart Green qPCR SuperMix (TransGen Biotech, Beijing, China). For all primer sets (S2 Table), the following cycling parameters were used: 95°C for 30 s followed by 40 cycles of 94°C for 15 s, 50°C for 30 s. For standardization of results, the relative abundance of 16S rRNA was used as the internal standard.

### Sensitivity assays for oxidative agents, antibiotics, heavy metals and heat treatment

Mid-exponential phase *Yptb* strains grown in YLB medium were collected, washed, and diluted 50-fold into M9 medium or PBS buffer with 0.4% glucose containing H_2_O_2_ (1.5 mM), CdCl_2_ (0.1 mM), Diamide (0.3 mM), or Gentamicin (0.2 μg/ml) and incubated at 26°C for 1 h, or subjected to heat shock (42°C) for 0.5 h. After treatment, the cultures were serially diluted and plated onto YLB agar plates, and colonies were counted after 20 h growth at 26°C. Percentage survival was calculated by dividing number of CFU of stressed cells by number of CFU of cells without stress [13]. All these assays were performed in triplicate at least three times.

### Fluorescence dye-based intracellular ROS detection

To detect intracellular ROS, the fluorescent reporter dye 3′-(p-hydroxyphenyl) fluorescein (HPF, Invitrogen), 5-(and-6)-chloromethyl-2′,7′-dichlorodihydrofluorescein diacetate, acetylester (CM-H2DCFDA, Life Technologies) and 2′,7′-dichlorodihydrofluorescein diacetate (H2DCFDA, Invitrogen) were used, as previously described [20]. Briefly, 1 ml samples were collected after treatment and then resuspended in 1 ml of PBS containing 10 μM HPF, CM-H2DCFDA or H2DCFDA, respectively. Samples were incubated in dark for 20 min. The cells were then pelleted, the supernatant removed, and were resuspended in 1 ml filtere-sterilized PBS. Two hundred microliters of the resultant cell suspension were transferred to a dark 96-well plate. Fluorescence signals were measured using a SpectraMax M2 Plate Reader (Molecular Devices) with excitation/emission wavelengths of 490/515 nm (HPF), 495/520 nm (CM-H2DCFDA and H2DCFDA). The results shown represented the mean of one representative assay performed in triplicate, and error bars represent standard deviation. Statistical analysis was carried out with Student’s *t*-test.

### Determination of intracellular ion content

Intracellular ion content was determined as described previously [58,59]. Briefly, cells were grown in YLB until mid-exponential phase. After 20 ml culture solutions were collected and washed with PBS for two times, the pellets were re-dissolved in 20 ml PBS buffer containing 0.4% glucose, 1.5 mM H_2_O_2_ and 1 μM Zn^2+^, and then incubated further for 20 min. These cultures were centrifuged at 4000 rpm for 10 min. The wet cell pellet weight was measured and bacteria were chemically lysed using Bugbuster (Novagen, Madison, WI) according to the manufacturer’s instructions. Bacteria were resuspended in Bugbuster solution by pipetting and incubation on a rotating mixer at a slow setting for 20 min. Total protein for each sample was measured by using NanoDrop ND-1000 spectrophotometer (NanoDrop Technologies) according to the manufacturer’s instructions. Each sample was diluted 100-fold in 2% molecular grade nitric acid to a total volume of 10 ml. Samples were analyzed by Inductively coupled plasma mass spectrometry (ICP-MS) (Varian 802-MS), and the results were corrected using the appropriate buffers for reference and dilution factors. Triplicate cultures of each strain were analyzed during a single experiment and the experiment was repeated at least three times.

### Zn^2+^ binding assays

Zn^**2+**^ binding was measured using isothermal titration calorimetry (ITC) at 25°C with a NANO-ITC 2G microcalorimeter (TA Instruments, USA). A control experiment in the absence of protein was performed to measure the heat generated due to Zn^**2+**^ dilution in the buffer. To obtain apo-protein, samples were dialyzed for 10 h at 4°C against 250 μM EDTA and 5 mM *o*-phenanthroline in 50 mM HEPES (pH 8.0), 150 mM NaCl, 15% glycerol, followed by three dialysis steps in 50 mM HEPES (pH 8.0), 150 mM NaCl, 15% glycerol to remove EDTA and *o*-phenanthroline. The dialysis buffer was used to prepare a 0.5 mM ZnSO_4_ solution used for titration. Protein concentrations in the sample solution were 40 μM. After a stable baseline was achieved the ZnSO_4_ titration was performed by a total of 25 injections of 5 μl into protein solutions (volume = 1.5 ml) until the protein sample was saturated with zinc. Blank titrations of the ZnSO_4_ solution into the dialysis buffer were performed to correct for the dilution heat of the zinc solution. Data reduction and analysis were performed with the Nano Analyze software (TA Instruments) fitting them to an independent binding model [60].

Zn^**2+**^ binding was also detected by using the Zn^2+^-binding dye 4-(2-pyridylazo)-resorcinol (PAR) [29]. Free PAR shows a peak absorbance at about 410 nm, shifting to 500 nm when PAR binds Zn^2+^. To determine if proteins binds Zn^2+^, increasing concentrations proteins (0–5 μM) were added to solutions containing 10 μM PAR and 5 μM Zn^2+^ in 50 mM HEPES (pH 8.0). A control experiment in the absence of protein was performed to obtain the spectra for free PAR bound to Zn^2+^, compared to the spectra gained in the presence of protein. A decrease in the absorbance at 500 nm, accompanied by an increase in the absorbance at 410 nm exhibits binding of Zn^2+^ by the protein.

### Iron-binding assay

Purified recombinant His_6_-Fur (Ypk_2991, ferric uptake regulator) and YezP proteins in 50 mM Tris-HCl (pH 7.4), 150 mM NaCl were incubated with 1 mM ferrous sulphate for 1 h at room temperature. The products were resolved on a 15% native PAGE. The gel was then stained with potassium ferricyanide solution (100 mM K_3_[Fe(CN)_6_] in 50 mM Tris-HCl, pH 7.4, 100 mM NaCl) for 10 min in the dark and destained with 10% trichloroacetic acid/methanol solution [61]. After taking an image of the stained gel, it was subjected to Coomassie blue staining using standard techniques. The iron binding protein Fur was used as a positive control.

### Macrophage infection and qRT-PCR

Bone marrow derived macrophages (BMDMs) from 6-week old C57BL/6 mice were prepared as previously described [62] and seeded in six-well multiplates at the density of 2x10^6^ cells per well. BMDMs were challenged with the indicated *Y*. *pseudotuberculosis* strains, which were cultured in YLB at 28°C for 20 hours before infection, at an MOI of 10. At the indicated time points, infected macrophages were washed 3 times with HBSS (hank’s balanced salt solution) to remove extracellular bacteria before being collected for total mRNA extraction using RNAqueous Total RNA Isolation Kit per manufacturer’s instruction. Indicated mRNA species were quantified using SYBR Green Real-Time PCR Master Mixes system (Life Technology), bacterial cells grown in YLB broth were used as controls. For standardization of results, the relative abundance of 16S rRNA was used as the internal standard.

### Mouse infections

All mice were maintained and handled in accordance with the animal welfare assurance policy issued by Northwest A&F University. Mid-exponential phase *Yptb* strains grown in YLB medium at 26°C, washed twice in sterilized PBS and used for orogastric infection of 6–8 weeks old female C57BL/6 mice using a ball-tipped feeding needle. For survival assays 3×10^9^ bacteria of each strain were applied to different groups of mice, and the survival rate of the mice was determined by monitoring the survival everyday for 21 days [63,64].

### Statistical analysis

Statistical analyses of survival assay, intracellular ion content determination, ROS determination and expression data were performed using paired two-tailed Student’s *t*-test. Survival times were analyzed using Kaplan-Meyer curves and comparisons were performed using the Log-Rank test. Statistical analyses were performed using GraphPad Prism Software (GraphPad Software, San Diego California USA).

### Accession numbers

Accession numbers for the genes described in this study in NCBI are: *yezP*, *ypk_3549; clpV4*, *ypk_3559*; *hcp4*, *ypk_3563*; *icmF4*, *ypk_3550*; *vgrG4*, *ypk_3558*; *hcp1*, *ypk_0385*; *hcp2*, *ypk_0803*; *hcp3*, *ypk_1481*; *oxyR*, *ypk_4079*; *znuB*, *ypk_2142*; *znuC*, *ypk_2141*; *fur*, *ypk_2991*; *katG*, *ypk_3388; sod*(*Fe/Mn*), *ypk_1863; sod*(*Cu/Zn*), *ypk_3445*.

## Supporting Information

S1 Table Bacterial strains and plasmids used in this study. (DOC)

S2 Table Primers used in this study. (DOC)

S1 Fig Identification of an OxyR binding site in the promoter region of T6SS-4. Putative OxyR binding site was indicated in the red box found by the online software Virtual Footprint. The OxyR-binding motif was also found in the region in red. The sequences used to replace the putative binding site are shown in blue below the promoter sequence. The ATG start codon of the first *orf* of T6SS-4 operon was marked in bold letters.
(TIF)

S2 Fig Detection the intracellular ROS in *Yptb* under oxidative condition. Oxidative stress induced the generation of intracellular ROS in T6SS-4 mutants. Intracellular ROS in mid-exponential phase bacteria exposed to H_2_O_2_ were stained with HPF (**A**), CM-H2DCFDA and H2DCFDA (**B**) dye, or without dye. Fluorescence was measured using a SpectraMax M2 Plate Reader (Molecular Devices) with excitation/emission wavelengths of 490/515 nm (HPF), 495/520 nm (CM-H2DCFDA and H2DCFDA). Data shown were the average of three independent experiments; error bars indicate SD from three independent experiments. **, p<0.01.
(TIF)

S3 Fig T6SS-4 is not involved in Mn^2+^ accumulation in *Yptb*. Mid-exponential phase of *Yptb* strains were exposed to1.5 mM H_2_O_2_ for 20 min in PBS containing 1 μM MnCl_2._ Mn^2+^ associated with bacterial cells was measured by inductively coupled plasmon resonance atomic absorption spectrometry (ICP-MS). Data shown were the average of three independent experiments; error bars indicate SD from three independent experiments.
(TIF)

S4 Fig Viability of *Yptb* strains under oxidative stress. Mid-exponential phase of *Yptb* strains were exposed to 1.5 mM H_2_O_2_ in PBS containing 1 μM ZnCl_2_ and the viability of the cells was determined at indicated time points. Data shown were the average of three independent experiments; error bars indicate SD from three independent experiments. **, *p*<0.01; n.s., not significant.
(TIF)

S5 Fig T6SS-4 and ZnuCB are only required for optimal growth under oxidative condition. Saturated bacterial cultures were diluted to an OD_600_ of 0.15 **A.** in YLB medium, **B.** YLB medium with 10 mM H_2_O_2_, **C.** YLB medium with 10 mM H_2_O_2_ and 100 μΜ TPEN, **D.** YLB medium with 10 mM H_2_O_2_ and 100 μΜ Zn^2+^, **E.** YLB medium with 10 mM H_2_O_2_, 100 μΜ TPEN and 100 μΜ Zn^2+^. The growth of the cultures was monitored by measuring OD_600_ at indicated time points. Data shown were the average of three independent experiments; error bars indicate SD from three independent experiments. ***, p<0.001; **, p<0.01; *, p<0.05.
(TIF)

S6 Fig Identification of a putative zinc finger motif in YezP (Ypk_3549). **A.** Identification of a predicted zinc finger in YezP by HHpred. Analysis was performed with the online software service (http://toolkit.tuebingen.mpg.de/hhpred/). **B.** The *yezP* gene localizes in the end of the T6SS-4 operon. **C.** Sequence alignment of the putative zinc finger motif in YezP with proteins containing experimentally verified zinc fingers. Conserved residues were marked in red.
(TIF)

S7 Fig Expression of *yezP* is regulated by OxyR. Total RNA was isolated from mid-exponential phase bacteria of indicated *Yptb* strains and the expression of *yezP* was evaluated by quantitative real-time PCR. Data shown were the average of three independent experiments; error bars indicate SD from three independent experiments. ***, *p*<0.001; **, *p*<0.01.
(TIF)

S8 Fig Binding of YezP to Zn^2+^ measured by PAR. Spectral scans of solutions containing 10 μM PAR without Zn^2+^ (black), with Zn^2+^ (brown) or with Zn^2+^ and increasing concentrations of recombinant YezP, YezP_H76A_ and control buffer (different color) are shown. Similar results were obtained in three independent experiments, and data shown are from one representative experiment done in triplicate.
(TIF)

S9 Fig YezP does not bind iron. **A.** Purified recombinant proteins analysis by 15% SDS-PAGE. M: protein marker. Lane 1: purified GST-YezP; Lane 2: GST-YezP treated with PreScission Protease. Lane 3: purified YezP. **B.** Iron binding analysis by 15% Native PAGE. Lane 1 and 2 showed the gel stained with Commassie bright blue, Lane 3 and 4 showed the same gel stained for iron by the potassium ferricynaide method. His_6_-Fur was used as positive control. Lane 1 and 3: His_6_-Fur; Lane 2 and 4: YezP. Similar results were obtained in three independent experiments, and data shown are from one representative experiment done in triplicate.
(TIF)

S10 Fig YezP is involved in Zn^2+^ accumulation in *Yptb*. Mid-exponential phase of *Yptb* strains were exposed to 1.5 mM H_2_O_2_ for 20 or 30 min in PBS containing 1 μM ZnCl_2_. Zn^2+^ associated with bacterial cells was determined by inductively coupled plasmon resonance atomic absorption spectrometry (ICP-MS). Data shown were the average of three independent experiments; error bars indicate SD from three independent experiments. **, *p*<0.01.
(TIF)

S11 Fig YezP secretion is induced under zinc limited conditions. **A.** *Yptb* wild-type strains expressing YezP-VSVG were grown in YLB or YLB with 100 μΜ TPEN, and the culture supernatant was detected by western blot. For the pellet fraction, the metabolic enzyme isocitrate dehydrogenase (ICDH) was detected as loading controls. Similar results were obtained in three independent experiments, and data shown are from one representative experiment done in triplicate. **B.** Relative secreted protein levels were quantified with Image Lab (Bio-Rad, USA). Data shown were the average of three independent experiments; error bars indicate SD from three independent experiments. *, *p*<0.05.
(TIF)
