# Supplementary material for: Type VI Secretion System Transports Zn2+ to Combat Multiple Stresses and Host Immunity
Source: PLoS Pathog. 2015 Jul 2;11(7):e1005020. doi: 10.1371/journal.ppat.1005020 (PMC4489752; doi:10.1371/journal.ppat.1005020)
Supplement: S1 Table — (DOC) [file ppat.1005020.s001.doc]

**S1 Table Bacterial strains and plasmids used in this study.**

| **Strain or plasmid** | **Relevant characteristics** | **Reference** |
| --- | --- | --- |
| ***E. coli*** |  |  |
| S17-1*λ pir* | *λ*-pir lysogen of S17-1, *thi pro hsdR hsdM^+^ recA* RP4 2-Tc::Mu-Km::Tn7 | [1] |
| BL21(DE3) | Host for expression vector pET28a | Novagen |
| XL1 Blue | Host for expression vector pGEX6p-1 | Novagen |
| JM109 | *recA1 supE44 endA1 hsdR17 gyrA96 relA1 thi* Δ(*lac-proAB*)F′(*traD36 proABlacI*^q^ *lacΔZM15*) | Stratagene |
| ***Y. pseudotuberculosis*** |  |  |
| *Yptb* WT | Wild-type *Y. pseudotuberculosis* pIB1, Nal^r^ | [2] |
| *Yptb*Δ*hcp4* | *hcp4* gene deleted in *Yptb*, Nal^r^ | [3] |
| *Yptb*Δ*clpV4* | *clpV4* gene deleted in *Yptb*, Nal^r^ | [4] |
| *Yptb*Δ*icmF4* | *icmF4* gene deleted in *Yptb*, Nal^r^ | This study |
| *Yptb*Δ*oxyR* | *oxyR* gene deleted in *Yptb*, Nal^r^ | This study |
| *Yptb*Δ*znuCB* | *znuCB* gene deleted in *Yptb*, Nal^r^ | This study |
| *Yptb*Δ*clpV4*Δ*znuCB* | *clpV4* and *znuCB* gene deleted in *Yptb*, Nal^r^ | This study |
| *Yptb*Δ*yezP* | *yezP* gene deleted in *Yptb*, Nal^r^ | This study |
| *Yptb*Δ*katG* | *katG* gene deleted in *Yptb*, Nal^r^ | This study |
| *Yptb*Δ*-sod(Cu/Zn)* | *sod(Cu/Zn)* gene deleted in *Yptb*, Nal^r^ | This study |
| *Yptb*Δ*-**sod(Fe/Mn)* | *sod(Fe/Mn)* gene deleted in *Yptb*, Nal^r^ | This study |
| *Yptb* WT(Vector) | *Yptb* Wild-type containing pKT100, Nal^r^, Km^r^ | [4] |
| *Yptb*Δ*clpV4*(Vector) | *Yptb*Δ*clpV4* containing pKT100, Nal^r^, Km^r^ | [4] |
| *Yptb*Δ*clpV4*(*clpV4*) | *Yptb*Δ*clpV4* containing pKT100-*clpV4*, Nal^r^, Km^r^ | [4] |
| *Yptb*Δ*clpV4*(*clpV4M*) | *Yptb*Δ*clpV4* containing pKT100-*clpV4M*, Nal^r^, Km^r^ | [4] |
| *Yptb*Δ*clpV4*(*yezP*) | *Yptb*Δ*clpV4* containing pKT100-*yezP*, Nal^r^, Km^r^ | This study |
| *Yptb*Δ*oxyR*(Vector) | *Yptb*Δ*oxyR* containing pKT100, Nal^r^, Km^r^ | This study |
| *Yptb*Δ*oxyR*(*oxyR*) | *Yptb*Δ*oxyR* containing pKT100-*oxyR*, Nal^r^, Km^r^ | This study |
| *Yptb*Δ*znuCB*(Vector) | *Yptb*Δ*znuCB* containing pKT100, Nal^r^, Km^r^ | This study |
| *Yptb*Δ*znuCB*(*znuCB*) | *Yptb*Δ*znuCB* containing pKT100-*znuCB*, Nal^r^, Km^r^ | This study |
| *Yptb*Δ*clpV4*Δ*znuC*B(Vector) | *Yptb*Δ*clpV4*Δ*znuCB* containing pKT100, Nal^r^, Km^r^ | This study |
| *Yptb*Δ*clpV4*Δ*znuCB*(*clpV4*) | *Yptb*Δ*clpV4*Δ*znuCB* containing pKT100-*clpV4*, Nal^r^, Km^r^ | This study |
| *Yptb*Δ*clpV4*Δ*znuCB*(*clpV4M*) | *Yptb*Δ*clpV4*Δ*znuCB* containing pKT100- *clpV4M*, Nal^r^, Km^r^ | This study |
| *YptbΔclpV4ΔznuCB(znuCB)* | *YptbΔclpV4ΔznuCB* containing pKT100-*znuCB*, Nal^r^, Km^r^ | This study |
| *Yptb*Δ*yezP*(Vector) | *Yptb*Δ*yezP* containing pKT100, Nal^r^, Km^r^ | This study |
| *Yptb*Δ*yezP*(*yezP*) | *Yptb*Δ*yezP* containing pKT100-*yezP*, Nal^r^, Km^r^ | This study |
| *Yptb*Δ*yezP*(*yezP_H76A_*) | *Yptb*Δ*yezP* containing pKT100-*yezP_H76A_*, Nal^r^, Km^r^ | This study |
| *Yptb* WT(pBS) | *Yptb* WT containing pBS-T6SS-4p, Nal^r^, Amp^r^ | [4] |
| *Yptb* WT(*vgrG4*GFP) | *Yptb* WT containing pBS-*vgrG4*GFP, Nal^r^, Amp^r^ | [4] |
| *Yptb* WT (*vgrG4*His_6_) | *Yptb* WT containing pBS*-vgrG4*His_6_, Nal^r^, Amp^r^ | [4] |
| **Plasmid** |  |  |
| pKT100 | Cloning vector, p15A replicon, Km^r^ | [5] |
| pKT100-*clpV4* | *clpV4* under the control of chloramphenicol resistance gene promoter in plasmid pKT100 | [4] |
| pKT100-*clpV4M* | *clpV4M* under the control of chloramphenicol resistance gene promoter in plasmid pKT100 | [4] |
| pKT100-*oxyR* | *oxyR* under the control of chloramphenicol resistance gene promoter in plasmid pKT100 | This study |
| pKT100-*zunCB* | *znuCB* under the control of chloramphenicol resistance gene promoter in plasmid pKT100 | This study |
| pKT100-*yezP* | *yezP* under the control of chloramphenicol resistance gene promoter in plasmid pKT100 | This study |
| pKT100-*yezP_H76A_* | *yezP_H76A_* under the control of chloramphenicol resistance gene promoter in plasmid pKT100 | This study |
| pET28a | Expression vector with N-terminal hexahistidine affinity tag, Km^r^ | Novagen |
| pET28a-*oxyR* | pET28a carrying *oxyR* coding region, Km^r^ | This study |
| pET28a-*fur* | pET28a carrying *fur* coding region, Km^r^ | This study |
| pGEX6p-1 | Expression vector with N-terminal GST tag, Amp^r^ | Novagen |
| pGEX6p-1-*yezP* | pGEX6p-1 carrying *yezP* coding region, Amp^r^ | This study |
| pGEX6p-1-*yezP_H76A_* | pGEX6p-1 carrying *yezP_H76A_* coding region, Amp^r^ | This study |
| pME6032 | Shuttle vector, Tc^r^ | [6] |
| pME6032-*yezP-vsvg* | pME6032 carrying *yezP-vsvg* coding region, Tc^r^ | This study |
| pME6032-*hcp4-vsvg* | pME6032 carrying *hcp4-vsvg* coding region, Tc^r^ | This study |
| pDM4 | Suicide vector, *mob*RK2, *ori*R6K, *pir*, *sacB*, Cm^r^ | [7] |
| pDM4-Δ*icmF4* | Construct used for in-frame deletion of *icmF4*, Cm^r^ | This study |
| pDM4-Δ*oxyR* | Construct used for in-frame deletion of *oxyR*, Cm^r^ | This study |
| pDM4-Δ*znuCB* | Construct used for in-frame deletion of *znuCB*, Cm^r^ | This study |
| pDM4-Δ*yezP* | Construct used for in-frame deletion of *yezP*, Cm^r^ | This study |
| pDM4-Δ*hcp1* | Construct used for in-frame deletion of *hcp1*, Cm^r^ | This study |
| pDM4-Δ*hcp2* | Construct used for in-frame deletion of *hcp2*, Cm^r^ | This study |
| pDM4-Δ*hcp3* | Construct used for in-frame deletion of *hcp3*, Cm^r^ | This study |
| pDM4-Δ*hcp4* | Construct used for in-frame deletion of *hcp4*, Cm^r^ | [3] |
| pDM4-Δ*katG* | Construct used for in-frame deletion of *katG*, Cm^r^ | This study |
| pDM4-Δ*sod(Fe/Mn)* | Construct used for in-frame deletion of *sod(Fe/Mn)*, Cm^r^ | This study |
| pDM4-Δ*sod(Fe/Mn)* | Construct used for in-frame deletion of *sod(Fe/Mn)*, Cm^r^ | This study |
| pDM4-*T6SS-1p::lacZ* | For *T6SS-1* promoter fusion to *Yptb*, Cm^r^ | [3] |
| pDM4-*T6SS-2p::lacZ* | For *T6SS-2* promoter fusion to *Yptb*, Cm^r^ | [3] |
| pDM4-*T6SS-3p::lacZ* | For *T6SS-3* promoter fusion to *Yptb*, Cm^r^ | [3] |
| pDM4-*T6SS-4p*::lacZ | For *T6SS-4* promoter fusion to *Yptb*, Cm^r^ | [3] |
| pDM4-*T6SS-4pM*::lacZ | *T6SS-4p*::lacZ, OxyR binding site mutated, Cm^r^ | This study |
| pDM4-*katGp*::lacZ | For *katG* promoter fusion to *Yptb*, Cm^r^ | This study |

*Nal^r^, Cm^r^, Km^r^, Tc^r^ and Amp^r^ represent resistance to naladixic acid, chloramphenicol, kanamycin, tetracycline and ampicillin at 15, 30, 50, 10 and 100 μg ml^-1^, respectively.

**References:**

1. Simon, R., Priefer, U. & Puhler, A. A broad host range mobilization system for in vivo genetic engineering: transposon mutagenesis in gram negative bacteria. Nature Biotechnology 1, 784–791. (1983).

2. Rosqvist, R., Skurnik, M. & Wolf-Watz, H. Increased virulence of *Yersinia pseudotuberculosis* by two independent mutations. Nature 334, 522–524. (1988).

3. Zhang, W. et al. Modulation of a thermoregulated type VI secretion system by AHL-dependent quorum sensing in *Yersinia pseudotuberculosis*. Arch Microbiol 193, 351-363. (2011).

4. Zhang, W. et al. A type VI secretion system regulated by OmpR in *Yersinia pseudotuberculosis* functions to maintain intracellular pH homeostasis. Environ Microbiol 15, 557-569. (2013).

5. Hu, Y., Lu, P., Wang, Y., Ding, L., Atkinson, S. & Chen, S. OmpR positively regulates urease expression to enhance acid survival of *Yersinia pseudotuberculosis*. Microbiology-SGM 155, 2522–2531. (2009).

6. Heeb, S., Blumer, C. & Haas, D. Regulatory RNA as mediator in GacA/RsmA-dependent global control of exoproduct formation in *Pseudomonas fluorescens* CHA0. J Bacteriol 184, 1046–1056. (2002).

7. Milton, D. L., O'Toole, R., Hörstedt, P. & Wolf-Watz, H. Flagellin A is essential for the virulence of *Vibrio anguillarum*. J Bacteriol 178, 1310–1319. (1996).
