# Supplementary material for: Type VI Secretion System Transports Zn2+ to Combat Multiple Stresses and Host Immunity
Source: PLoS Pathog. 2015 Jul 2;11(7):e1005020. doi: 10.1371/journal.ppat.1005020 (PMC4489752; doi:10.1371/journal.ppat.1005020)
Supplement: S2 Table — (DOC) [file ppat.1005020.s002.doc]

**S2 Table**  Primers used in this study.

| **Primers** | **5’-3’ sequence** | **Function** |
| --- | --- | --- |
| *oxyR-*1F*-BamH*I | GTGCGGATCCTTCCAGATCGTCGCTGCC | To generate pDM4-Δ*oxyR* |
| *oxyR-*1R | CAGCCAACGCCACTAAGTAC |  |
| *oxyR-*2F | **GTACTTAGTGGCGTTGGCTG**GCAAGAGCGCATGGCCCCAT |  |
| *oxyR-*2R*-Sal*I | CTGCGTCGACAGCCACAGCACGTCATTATT |  |
| *oxyR-*F*-Sph*I | GTGCGCATGCATGAATATTCGTGATCTAGA | To generate pKT100-*oxyR* |
| *oxyR-*F*-BamH*I | GTGCGGATCCATGAATATTCGTGATCTAGA | To generate pET28a-*oxyR* |
| *oxyR-*R*-Sal*I | GTGCGTCGACTTAAATCGCCTGTTCTAATG | To generate pKT100-*oxyR*, pET28a-*oxyR* |
| *znuCB-*1F*-BamH*I | CTGAGGATCCAGCATTAATGACCGCTGGCC | To generate pDM4-Δ*znuCB* |
| *znuCB-*1R | AGTACCCGGCGAGAGCCAA |  |
| *znuCB-*2F | **TTGGCTCTCGCCGGGTACT**GTGCTGCCGCCATGTTTGT |  |
| *znuCB-*2R*-Sal*I | GTGCGTCGACTTCAGGCAGCAAAACAACGC |  |
| *znuCB-*F*-Sph*I | GTGCGCATGCATGATGCCCATATTGGTAACGC | To generate pKT100-*znuCB* |
| *znuCB-*R*-Sal*I | GTGCGTCGACCTATCCTCGTGCTTTTTGCGAC |  |
| *3549-*1F*-Bgl*II | GTGAAGATCTGCCAGAGAAAGCGGTGGAGC | To generate pDM4-Δ*yezP* |
| *3549-*1R | TCGCGGTTAGAGATTCTTAGGAC |  |
| *3549-*2F | **TCCTAAGAATCTCTAACCGCGA**GGTTTCCCAGAGGAAATGAAAAGT |  |
| *3549-*2R*-Sal*I | GTGCGTCGACGATATTGTCGGGATCAACCAGG |  |
| *3549-*F*-Sph*I | CTGAGCATGCATGCTAGATCCTAAAAACATCCAGA | To generate pKT100-*yezP* |
| *3549-*F*-BamH*I | CTGAGGATCCATGCTAGATCCTAAAAACATCCAGA | To generate pGEX6p-1-*yezP* |
| *3549-*R*-Sal*I | GTGCGTCGACCTACATTTTGGGCAATTTATCAAAC | To generate pKT100-*yezP*, pGEX6p-1-*yezP* |
| *fur-F-BamH*I | CTGAGGATCCATGACTGACAACAACAAAGCCT | pET28a-*fur* |
| *fur-R-Sal*I | GTGCGTCGACTTATCTTTTACTGTGTGCAGACTCA |  |
| *icmF4-*1F*-BamH*I | CGCGGATCCAGGCTCCTACAACCAAAGTC | To generate pDM4-Δ*icmF4* |
| *icmF4 -*1R | TTGGGTATTCGTTCCTTGAA |  |
| *icmF4 -*2F | **TTCAAGGAACGAATACCCAA**AAGCATTGGCACAGTTCTCA |  |
| *icmF4 -*2R*-Sal*I | ACGCGTCGACATCGCAACGGCACTAACCTG |  |
| *hcp1-*1F*-Xba*I | ACGCTCTAGAGCATATCTTCATTGCCAAGC | To generate pDM4-Δ*hcp1* |
| *hcp1-*1R | AATATTGCCCTGCGTTTTCC |  |
| *hcp1-*2F | **GGAAAACGCAGGGCAATATT**ACCTCTGGTGCTGATGACTG |  |
| *hcp1-*2R*-Xho*I | CCGCTCGAGTGTCGATAAGGCGCTGAAAT |  |
| *hcp2-*1F*-BamH*I | CGCGGATCCAGGCACCCAATGCGGATGTT | To generate pDM4-Δ*hcp2* |
| *hcp2-*1R | CGTCGTCTTTCAGCCACAGATA |  |
| *hcp2-*2F | **TATCTGTGGCTGAAAGACGACG**TTCCGATTCTTGGAATGAGC |  |
| *hcp2-*2R*-Xho*I | CCGCTCGAGGACATCGAGGGTAAATTTATCCAG |  |
| *hcp3-*1F*-BamH*I | CGCGGATCCGTCTGCCTTATGGGATTAATTC | To generate pDM4-Δ*hcp3* |
| *hcp3-*1R | GCCAAACATCGAGTGTAGGA |  |
| *hcp3-*2F | **TCCTACACTCGATGTTTGGC**CTGCTAACTACGATGTTAAAGC |  |
| *hcp3-*2R*-Xho*I | CCGCTCGAGGCAAATCCCCAAAAGAATG |  |
| *3549H76AF* | **AATGTTGATGCTGCCCAGAAAATGCAA**AGTGAGGA | To generate yezP_H76A_ |
| *3549H76AR* | **TTGCATTTTCTGGGCAGCATCAACATT**GTCCTGC |  |
| *3549-*F*-EcoR*I | TCCGGAATTCATGCTAGATCCTAAAAACATCCAGA | To generate pME6032-*yezP-vsvg* |
| *3549taa-*R*-VSVG-Bgl*II | GTGAAGATCTTCATTTTCCTAATCTATTCATTTCAATATCTGTATACATTTTGGGCAATTTATCAAACATA |  |
| *hcp4-*F*-EcoR*I | TCCGGAATTCATGGCAGCTTTAGTCGATTACTT | To generate pME6032-*hcp4-vsvg* |
| *hcp4taa-*R*-VSVG-Bgl*II | GTGAAGATCTTCATTTTCCTAATCTATTCATTTCAATATCTGTATAAATACGGTTGTTCAATTTCAGATC |  |
| *pvgrG4-*F | AGGGAATCCATCCTACCA | qRT-PCR |
| *pvgrG4-*R | AATTTGTCTTGCCGTTGC |  |
| *phcp4-*F | GTAACTGTCTGGTGTCCTCC |  |
| *phcp4-*R | CCATCAGGTTGCTGCTCT |  |
| *pclpV4-*F | GGCGTCACCTTCTCCTATC |  |
| *pclpV4-*R | TGAACCTCGCTGGTCTGT |  |
| *pyezP-*F | GAGCGATATGAATAAGATATTGGG |  |
| *pyezP-*R | TGGAGAGTGTGCTGCGAAG |  |
| *16S RNA-*F | CTAGCGATTCCGACTTCAT |  |
| *16S RNA-*R | CCCTTATCCTTTGTTGCC |  |
| *pkatG*-F | TGACCGTGCCTGAAATGA |  |
| *pkatG*-R | TAAGCTGCCCTGGCGTAT |  |
| T6p4 footprinting-F | AGAAGGCGTTGATGTTTGAC | OxyR Footprinting |
| T6p4 footprinting-R | AACGCCGAATAATGCTTGAG |  |
| T6p-oxyR-F-5'biotin | GTGGATTTCGCCTCAGGCAT | EMSA |
| T6p-oxyR-R-5'biotin | CATCCTGATTTACATACCTG |  |
| T6p-oxyR-F | GTGGATTTCGCCTCAGGCAT |  |
| T6p-oxyR-R | CATCCTGATTTACATACCTG |  |
| T6SS-4pM-F | **TGGGTCTCTGTTGTGCACG**GGCTTGCTGGGTATGTCTTTATTTTTTTCTTATTTAAG | OxyR binding site replacement |
| T6SS-4pM-R | **CGTGCACAACAGAGACCCA**GCAAGCCCGTGAACAGGTATGTAAATCAGGATGATTAT |  |
| T6SS-4p-*Sal*I-F | CTCGGTCGACTAGGTTGGACATATCTCCCCATGT |  |
| T6SS-4p-*Xba*I-R | CTCGTCTAGACATAACGCCGAATAATTGCTT |  |
| *katG*-1F-*Bgl*II | GTGAAGATCTTTCAATATATTTACCTTTGCCC | To generate pDM4*-ΔkatG* |
| *katG-1R* | CGTAGGTGTATTATGTACAATGG |  |
| *katG-2F* | **CCATTGTACATAATACACCTACG**CTAAATAGAGGGGAGGATTTATC |  |
| *katG-2R-Sal*I | ACGCGTCGACTATCCGCCAGATCAAAATTC |  |
| *katG*-p-*Sal*I-F | ATGCGTCGACGCGGTAAGACAGGATTTCA | To generate pDM4-*katG-p::lacZ* |
| *katG*-p-*Xba*I-R | ATGCTCTAGACATGATTTTTTTTAACATATATAAGTCCCC |  |
| *sod(Cu/Zn)*-1F-*BamH*I | CTGAGGATCCTGGCTGTTATCGCTGAAGCC | To generate pDM4*-Δsod(Cu/Zn)* |
| *sod(Cu/Zn)*-1R | TCCCTTATATGTTGATTAAGTCTGG |  |
| *sod(Cu/Zn)*-2F | **CCAGACTTAATCAACATATAAGGGA**TGTGGAGTCATTGAGTAACCGT |  |
| *sod(Cu/Zn)*-2R-*Sal*I | GTGCGTCGACTGCCAGTGAGTGAGCACAGC |  |
| *sod(Fe/Mn)*-1F-*Bgl*II | GTGAAGATCTAGGCAACAATAAGACGAGTAC | To generate pDM4*-Δsod(Fe/Mn)* |
| *sod(Fe/Mn)*-1R | GTAAGGTAATGCAGGTAATTCA |  |
| *sod(Fe/Mn)*-2F | **TGAATTACCTGCATTACCTTAC**TGGTCTTTCGCTGAGAAAA |  |
| *sod(Fe/Mn)*-2R-*Sal*I | ACGCGTCGACGATAGAGTCAGTAAAATCGCCA |  |

Underlined sites indicate restriction enzyme cutting sites added for cloning. Letters in boldface denote the annealing regions for overlap PCR.
