# Supplementary figures and images for: Type VI Secretion System Transports Zn2+ to Combat Multiple Stresses and Host Immunity
Source: PLoS Pathog. 2015 Jul 2;11(7):e1005020. doi: 10.1371/journal.ppat.1005020 (PMC4489752; doi:10.1371/journal.ppat.1005020)

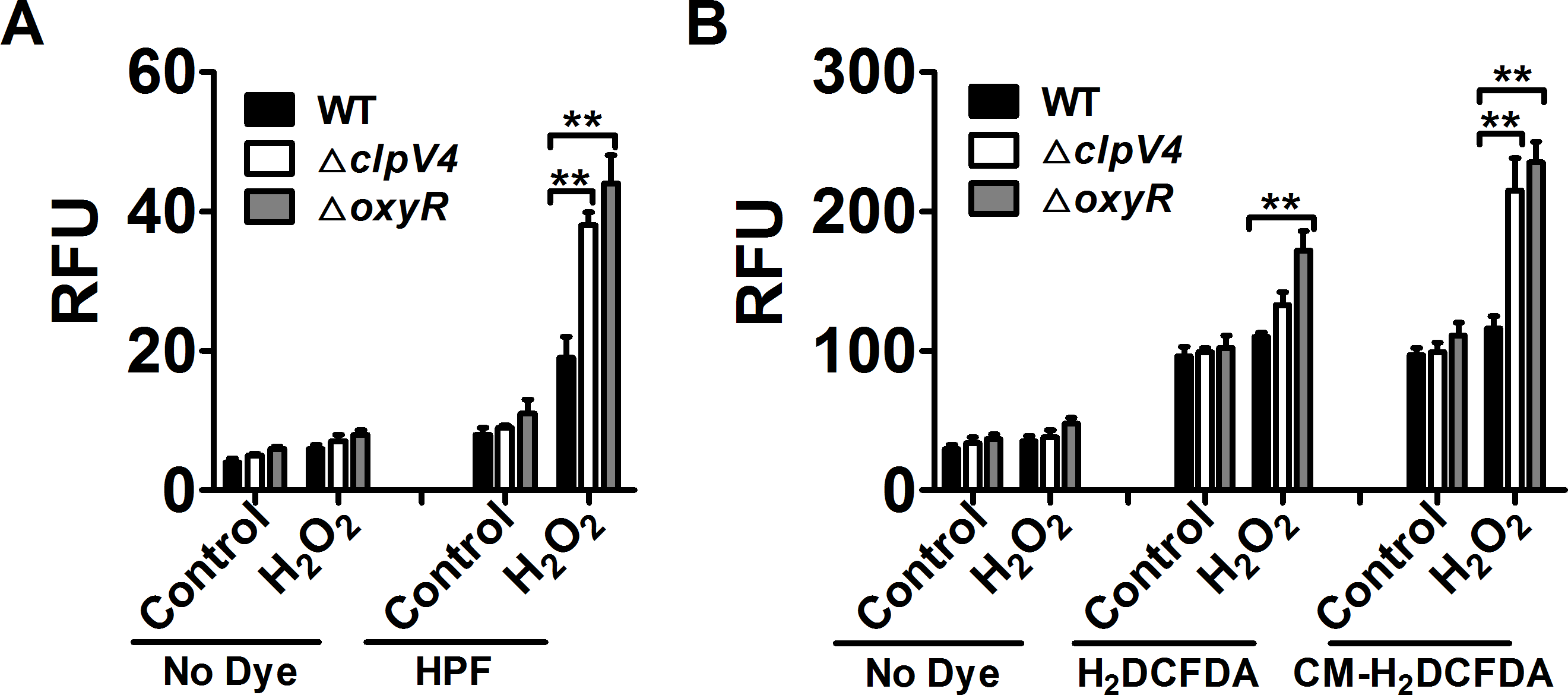

Supplement: S2 Fig — Oxidative stress induced the generation of intracellular ROS in T6SS-4 mutants. Intracellular ROS in mid-exponential phase bacteria exposed to H2O2 were stained with HPF (A), CM-H2DCFDA and H2DCFDA (B) dye, or without dye. Fluorescence was measured using a SpectraMax M2 Plate Reader (Molecular Devices) with excitation/emission wavelengths of 490/515 nm (HPF), 495/520 nm (CM-H2DCFDA and H2DCFDA). Data shown were the average of three independent experiments; error bars indicate SD from three independent experiments. **, p<0.01. (TIF) [file ppat.1005020.s004.tif]

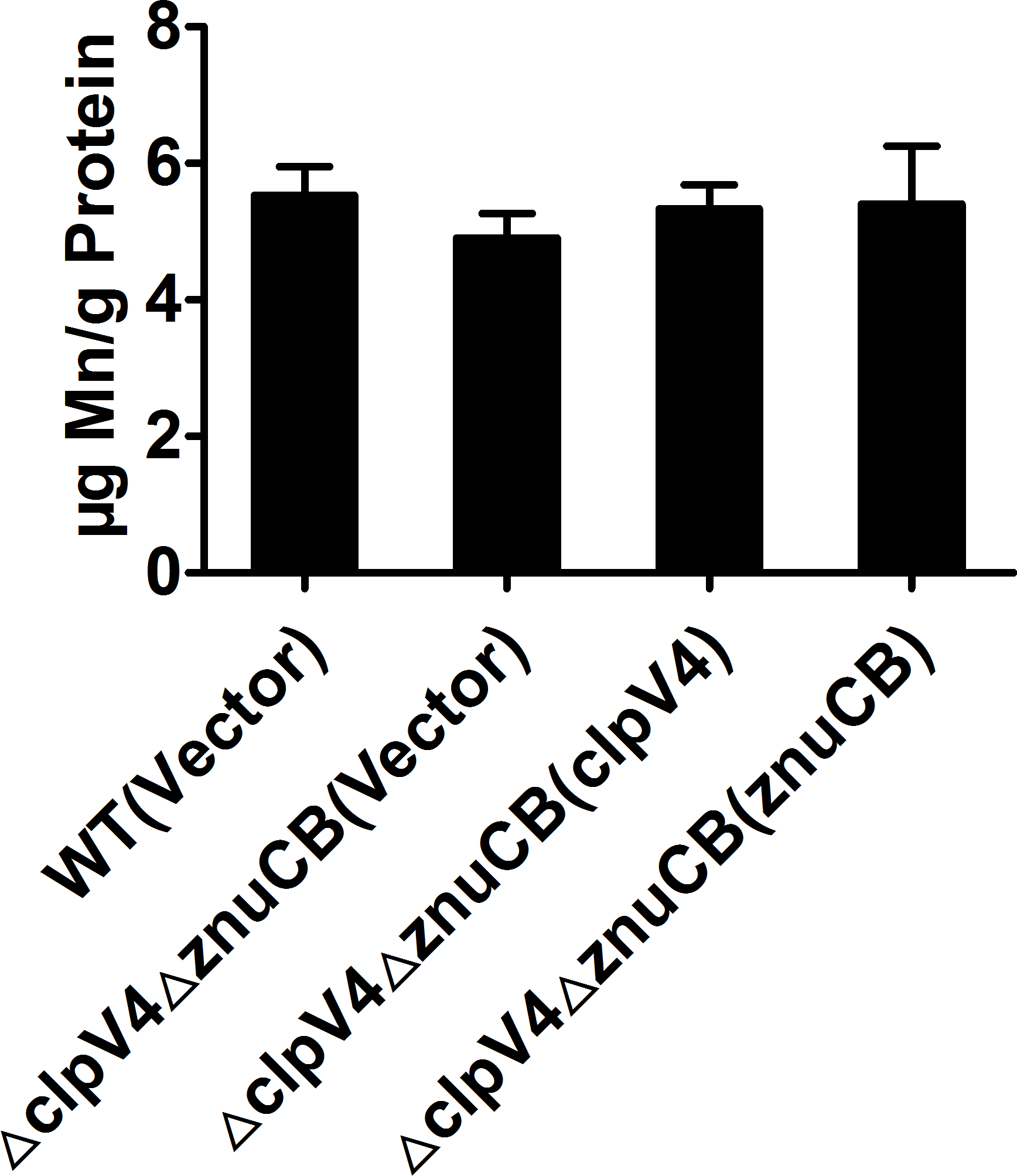

Supplement: S3 Fig — Mid-exponential phase of Yptb strains were exposed to1.5 mM H2O2 for 20 min in PBS containing 1 μM MnCl2. Mn2+ associated with bacterial cells was measured by inductively coupled plasmon resonance atomic absorption spectrometry (ICP-MS). Data shown were the average of three independent experiments; error bars indicate SD from three independent experiments. (TIF) [file ppat.1005020.s005.tif]

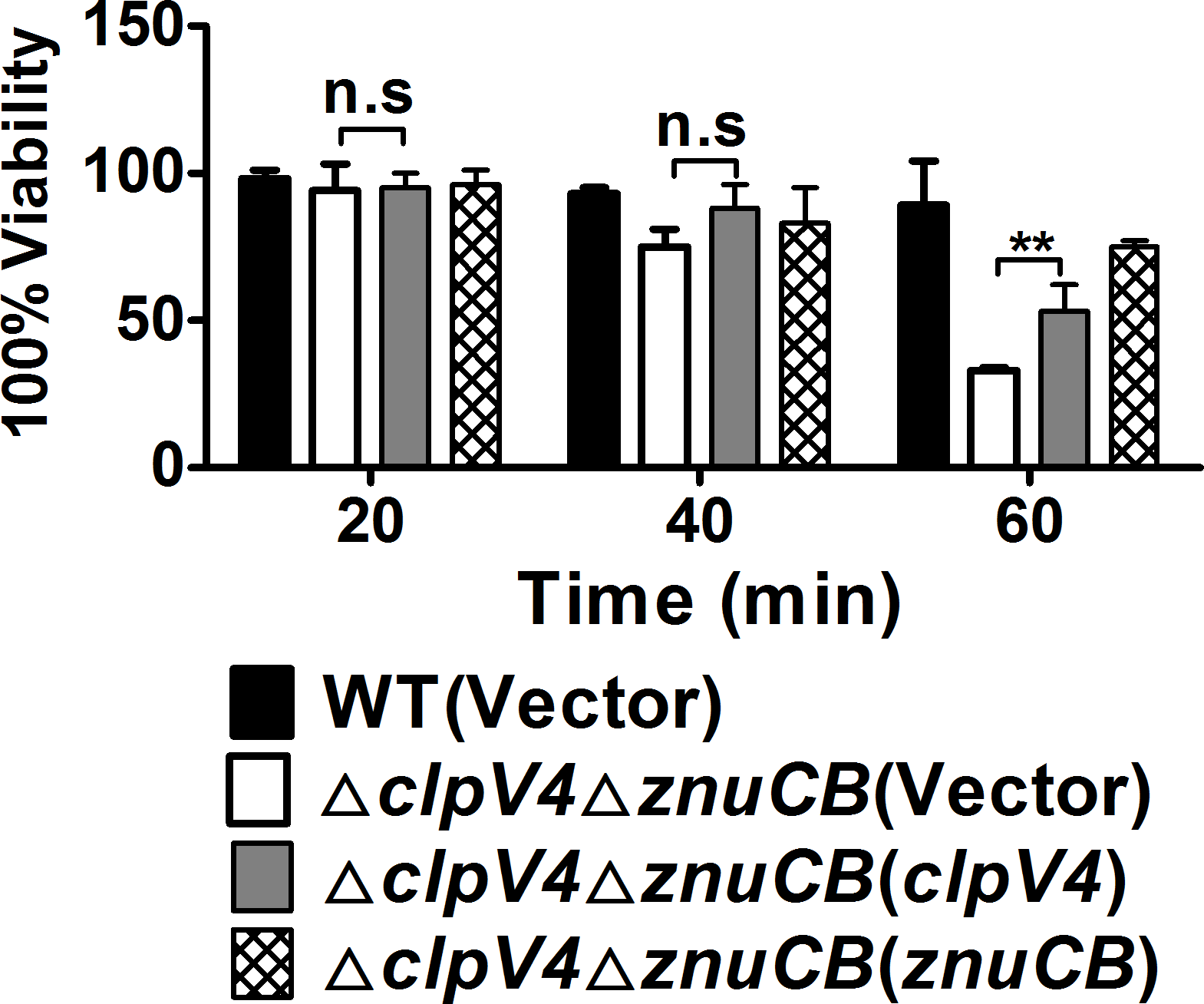

Supplement: S4 Fig — Mid-exponential phase of Yptb strains were exposed to 1.5 mM H2O2 in PBS containing 1 μM ZnCl2 and the viability of the cells was determined at indicated time points. Data shown were the average of three independent experiments; error bars indicate SD from three independent experiments. **, p<0.01; n.s., not significant. (TIF) [file ppat.1005020.s006.tif]

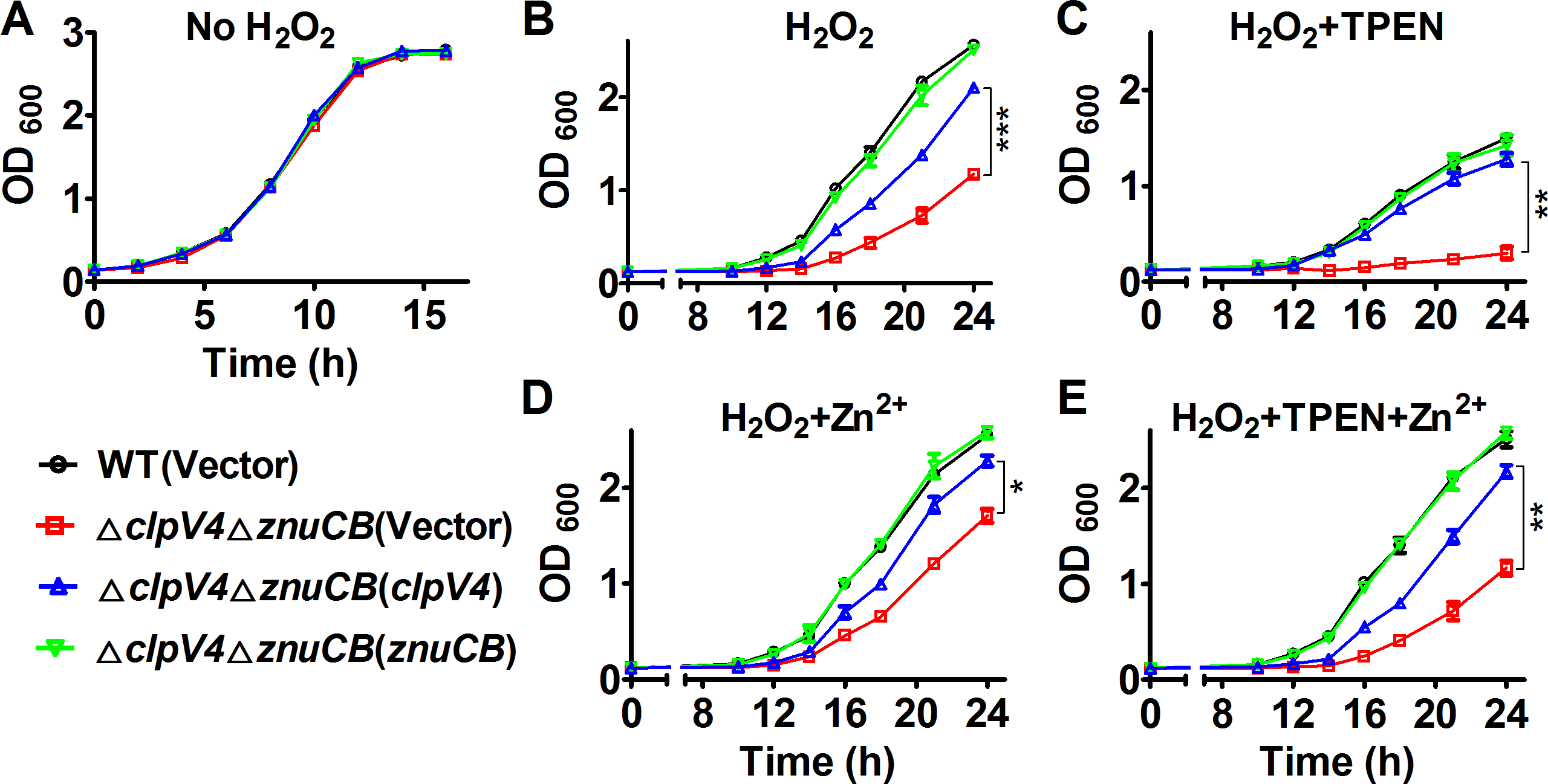

Supplement: S5 Fig — Saturated bacterial cultures were diluted to an OD600 of 0.15 A. in YLB medium, B. YLB medium with 10 mM H2O2, C. YLB medium with 10 mM H2O2 and 100 μΜ TPEN, D. YLB medium with 10 mM H2O2 and 100 μΜ Zn2+, E. YLB medium with 10 mM H2O2, 100 μΜ TPEN and 100 μΜ Zn2+. The growth of the cultures was monitored by measuring OD600 at indicated time points. Data shown were the average of three independent experiments; error bars indicate SD from three independent experiments. ***, p<0.001; **, p<0.01; *, p<0.05. (TIF) [file ppat.1005020.s007.tif]

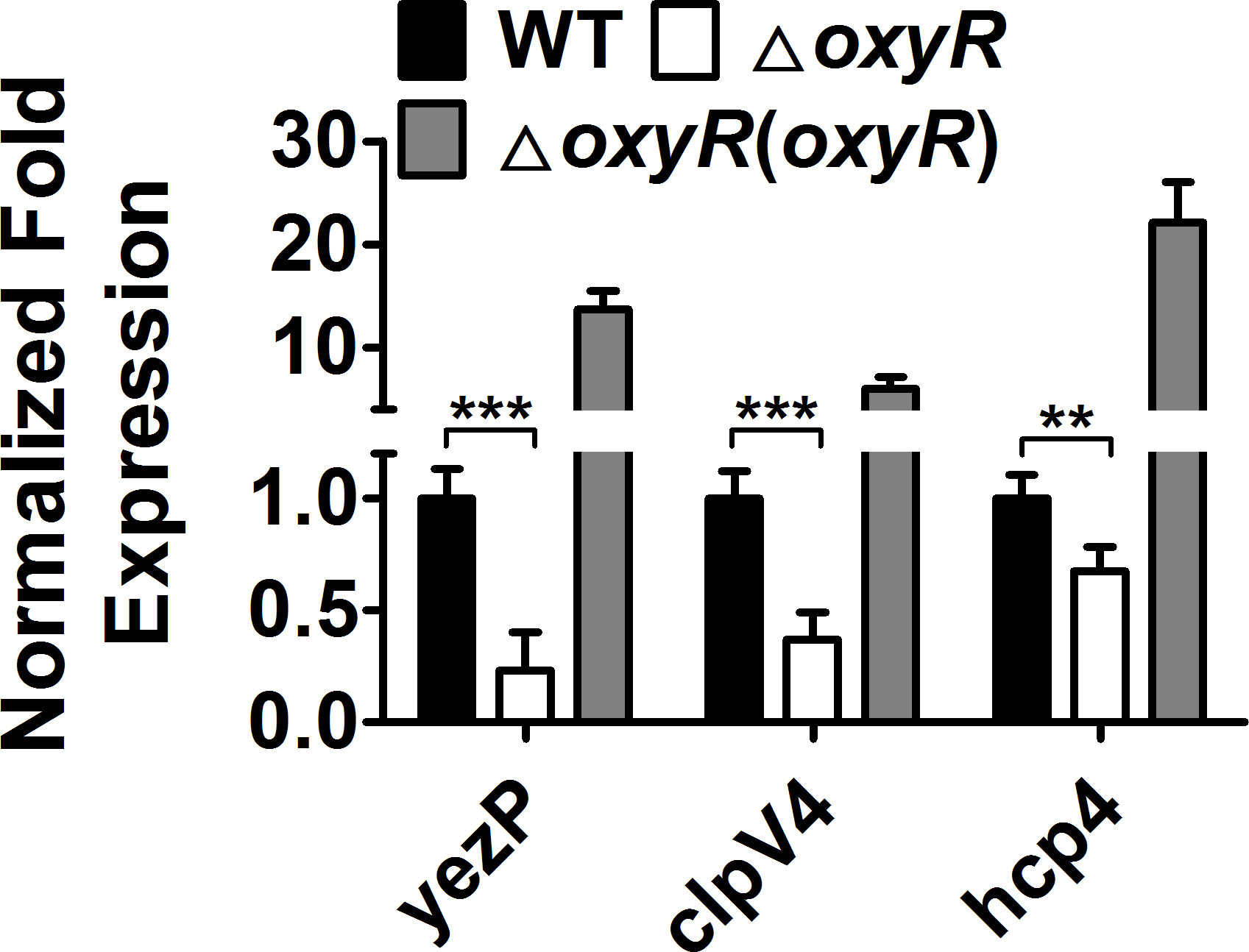

Supplement: S7 Fig — Total RNA was isolated from mid-exponential phase bacteria of indicated Yptb strains and the expression of yezP was evaluated by quantitative real-time PCR. Data shown were the average of three independent experiments; error bars indicate SD from three independent experiments. ***, p<0.001; **, p<0.01. (TIF) [file ppat.1005020.s009.tif]

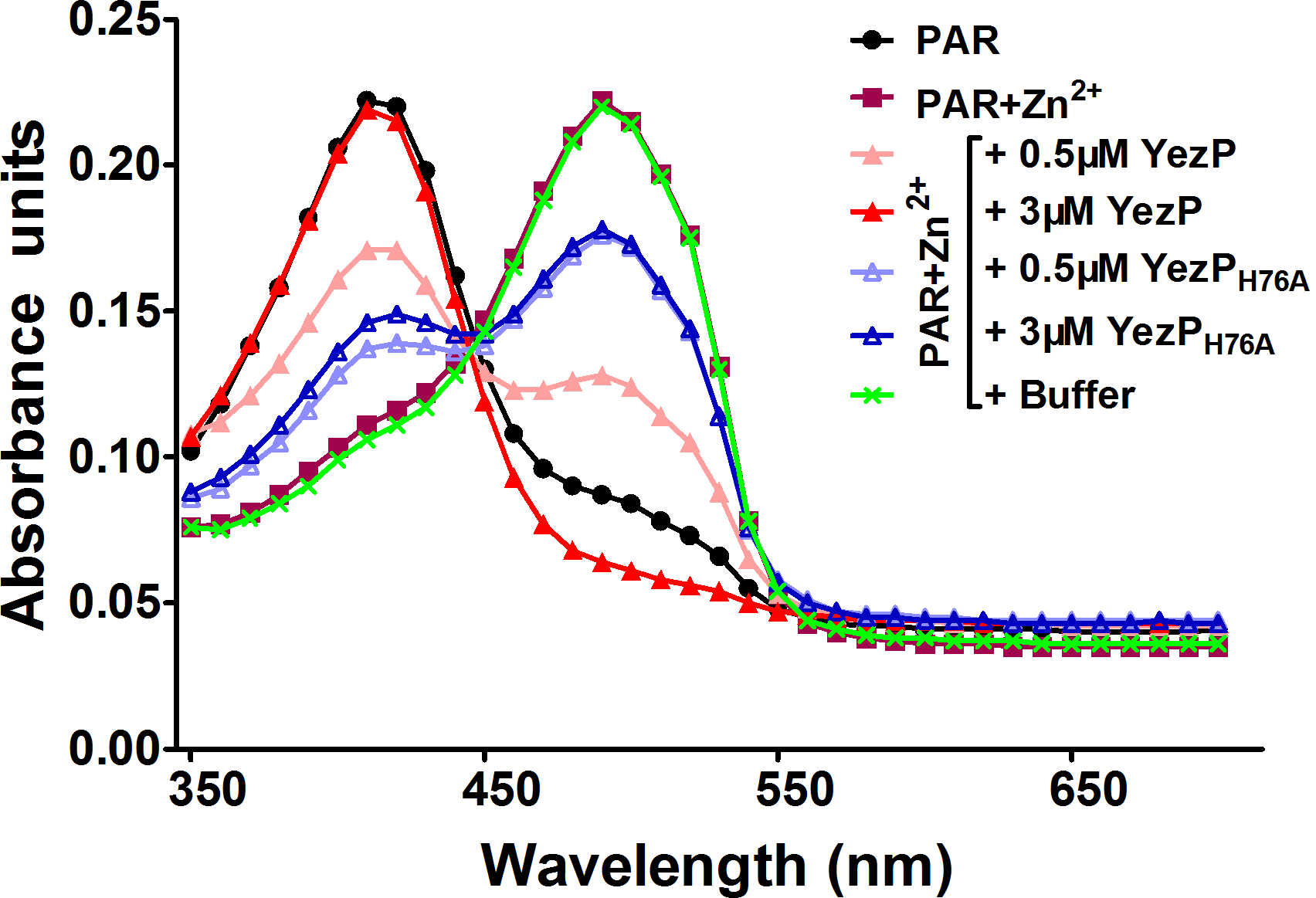

Supplement: S8 Fig — Spectral scans of solutions containing 10 μM PAR without Zn2+ (black), with Zn2+ (brown) or with Zn2+ and increasing concentrations of recombinant YezP, YezPH76A and control buffer (different color) are shown. Similar results were obtained in three independent experiments, and data shown are from one representative experiment done in triplicate. (TIF) [file ppat.1005020.s010.tif]

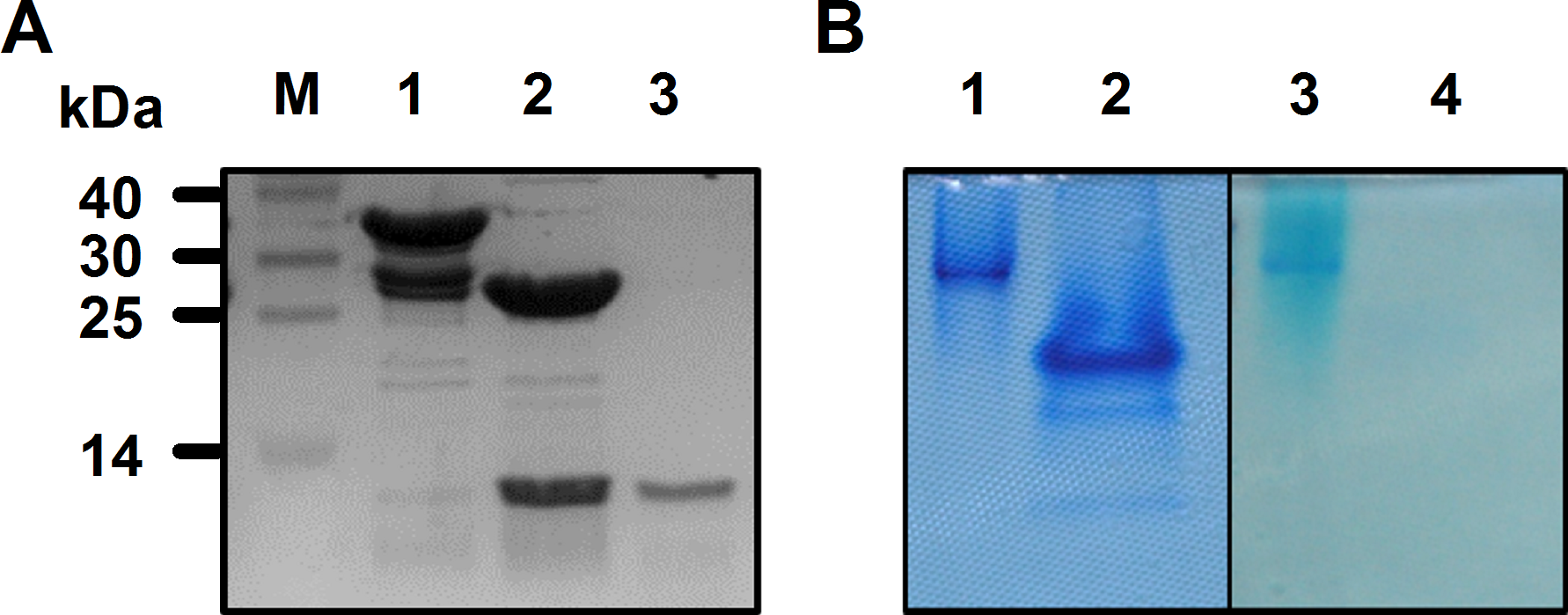

Supplement: S9 Fig — A. Purified recombinant proteins analysis by 15% SDS-PAGE. M: protein marker. Lane 1: purified GST-YezP; Lane 2: GST-YezP treated with PreScission Protease. Lane 3: purified YezP. B. Iron binding analysis by 15% Native PAGE. Lane 1 and 2 showed the gel stained with Commassie bright blue, Lane 3 and 4 showed the same gel stained for iron by the potassium ferricynaide method. His6-Fur was used as positive control. Lane 1 and 3: His6-Fur; Lane 2 and 4: YezP. Similar results were obtained in three independent experiments, and data shown are from one representative experiment done in triplicate. (TIF) [file ppat.1005020.s011.tif]

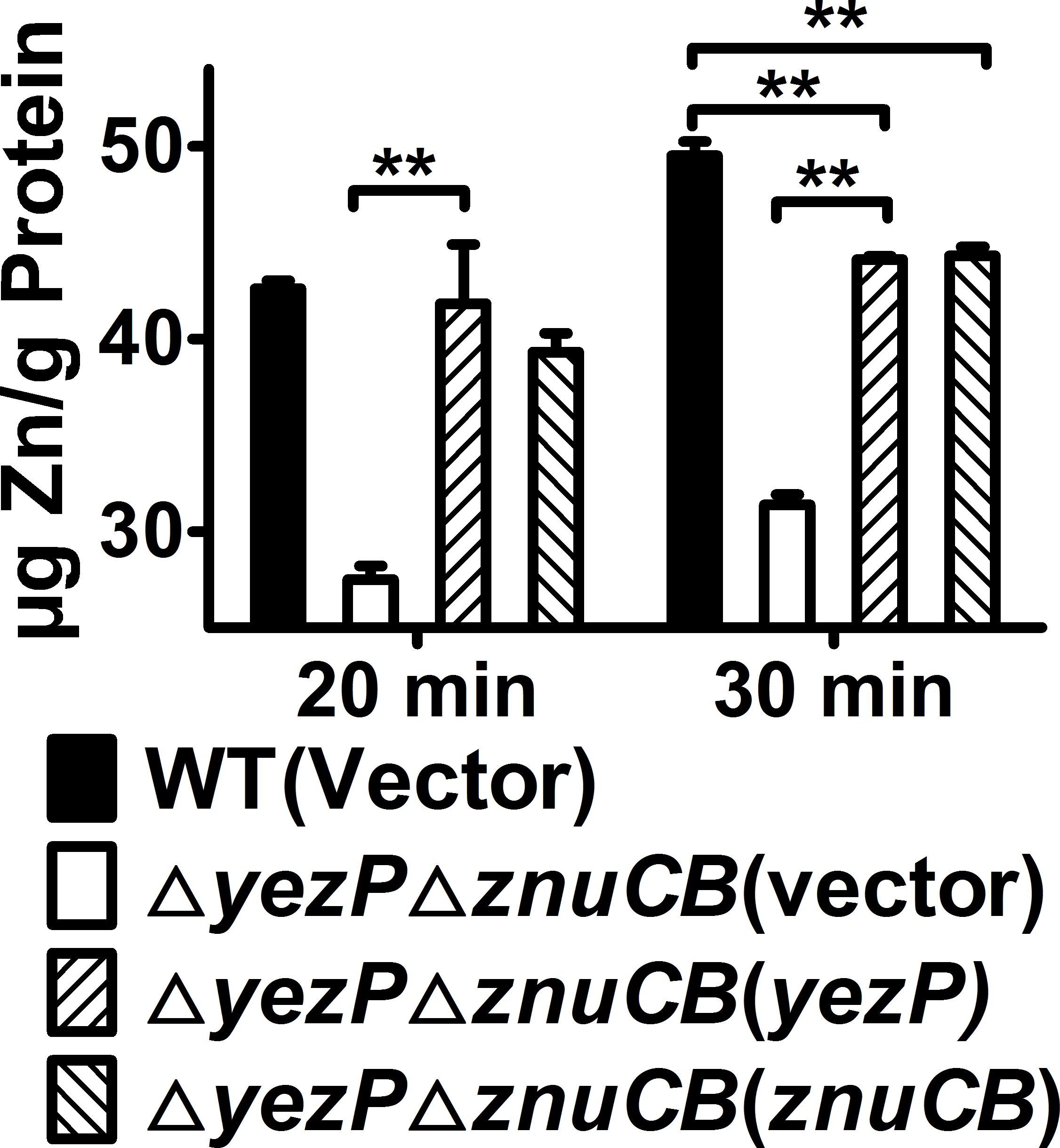

Supplement: S10 Fig — Mid-exponential phase of Yptb strains were exposed to 1.5 mM H2O2 for 20 or 30 min in PBS containing 1 μM ZnCl2. Zn2+ associated with bacterial cells was determined by inductively coupled plasmon resonance atomic absorption spectrometry (ICP-MS). Data shown were the average of three independent experiments; error bars indicate SD from three independent experiments. **, p<0.01. (TIF) [file ppat.1005020.s012.tif]

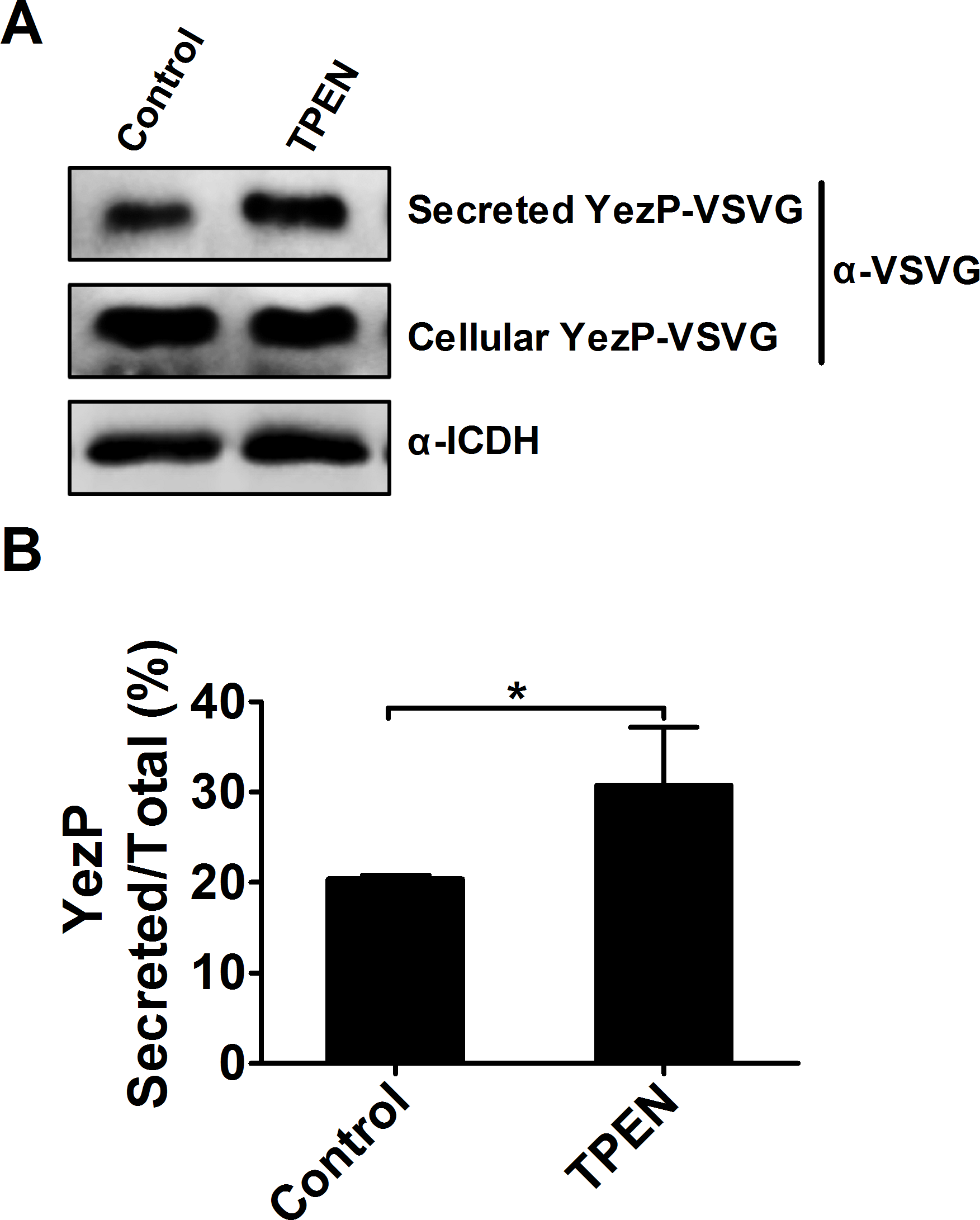

Supplement: S11 Fig — A. Yptb wild-type strains expressing YezP-VSVG were grown in YLB or YLB with 100 μΜ TPEN, and the culture supernatant was detected by western blot. For the pellet fraction, the metabolic enzyme isocitrate dehydrogenase (ICDH) was detected as loading controls. Similar results were obtained in three independent experiments, and data shown are from one representative experiment done in triplicate. B. Relative secreted protein levels were quantified with Image Lab (Bio-Rad, USA). Data shown were the average of three independent experiments; error bars indicate SD from three independent experiments. *, p<0.05. (TIF) [file ppat.1005020.s013.tif]
